# *C*. *elegans* DAF-16/FOXO interacts with TGF-ß/BMP signaling to induce germline tumor formation via mTORC1 activation

**DOI:** 10.1371/journal.pgen.1006801

**Published:** 2017-05-26

**Authors:** Wenjing Qi, Yijian Yan, Dietmar Pfeifer, Erika Donner v. Gromoff, Yimin Wang, Wolfgang Maier, Ralf Baumeister

**Affiliations:** 1 Bioinformatics and Molecular Genetics, Faculty of Biology, University of Freiburg, Freiburg, Baden-Wuerttemberg, Germany; 2 Department of Internal Medicine, University Medical Center Freiburg, Freiburg, Baden-Wuerttemberg, Germany; 3 Center for Biochemistry and Molecular Cell Research, Faculty of Medicine, University of Freiburg, Freiburg, Baden-Wuerttemberg, Germany; 4 Centre for Biological Signaling Studies (BIOSS), University of Freiburg, Freiburg, Baden-Wuerttemberg, Germany; Stanford University Medical Center, UNITED STATES

## Abstract

Activation of the FOXO transcription factor DAF-16 by reduced insulin/IGF signaling (IIS) is considered to be beneficial in *C*. *elegans* due to its ability to extend lifespan and to enhance stress resistance. In the germline, cell-autonomous DAF-16 activity prevents stem cell proliferation, thus acting tumor-suppressive. In contrast, hypodermal DAF-16 causes a tumorous germline phenotype characterized by hyperproliferation of the germline stem cells and rupture of the adjacent basement membrane. Here we show that cross-talk between DAF-16 and the transforming growth factor ß (TGFß)/bone morphogenic protein (BMP) signaling pathway causes germline hyperplasia and results in disruption of the basement membrane. In addition to activating MADM/NRBP/*hpo-11* gene alone, DAF-16 also directly interacts with both R-SMAD proteins SMA-2 and SMA-3 in the nucleus to regulate the expression of mTORC1 pathway. Knocking-down of BMP genes or each of the four target genes in the hypodermis was sufficient to inhibit germline proliferation, indicating a cell-non-autonomously controlled regulation of stem cell proliferation by somatic tissues. We propose the existence of two antagonistic DAF-16/FOXO functions, a cell-proliferative somatic and an anti-proliferative germline activity. Whereas germline hyperplasia under reduced IIS is inhibited by DAF-16 cell-autonomously, activation of somatic DAF-16 in the presence of active IIS promotes germline proliferation and eventually induces tumor-like germline growth. In summary, our results suggest a novel pathway crosstalk of DAF-16 and TGF-ß/BMP that can modulate mTORC1 at the transcriptional level to cause stem-cell hyperproliferation. Such cell-type specific differences may help explaining why human FOXO activity is considered to be tumor-suppressive in most contexts, but may become oncogenic, e.g. in chronic and acute myeloid leukemia.

## Author summary

The transcription factor FOXO is a well-known tumor suppressor whose activity is controlled by nutrients and stress signaling. In the roundworm *C*. *elegans*, the activity of the FOXO protein DAF-16 is best known for its beneficial role in stress response and long lifespan. However, FOXO proteins may also promote tumor cell growth and maintenance in chronic and acute myeloid leukemia, suggesting that may have different roles in distinct contexts. Previously we have shown that selective activation of DAF-16 in the epidermis causes a tumorous growth in the stem cells of the *C*. *elegans* germline. Now we demonstrate that this oncogenic activity of DAF-16 is mediated by interactions with the transforming growth factor (TGFß)/Bone Morphogenic protein (BMP) signaling pathway. In the epidermis, direct binding of DAF-16 and R-SMAD proteins of the BMP pathway helps to activate genes involved in the mTORC1 signaling pathway that is frequently activated in tumors. We propose that the transcription factor DAF-16/FOXO may be controlled in different ways in the stem cells, in which its activity normally prevents tumor formation, and in other tissues, in which defects in controlling its activity may result in overwriting the beneficial stem cell activity to eventually promote tumor cell growth.

## Introduction

The *C*. *elegans* FOXO transcription factor DAF-16 is one of the most intensively studied transcription factors due to its ability to extend lifespan and confer stress resistance [1]. In well-fed and stress-free animals, DAF-16 is inactivated by the insulin/IGF-1 signaling (IIS) through the IIS receptor DAF-2, phosphatidylinositol-4,5-bisphosphate 3-kinase (PI3K) and the AGC kinases AKT-1, AKT-2, and SGK-1 that promote its cytosolic localization [2–4]. Upon reduced IIS or stress stimuli DAF-16 becomes nuclearly localized and transcriptionally active. Active DAF-16 extends lifespan and increases stress resistance, indicating an overall beneficial effect of DAF-16 activation. DAF-16 activity in the germline, the source of stem cells in *C*. *elegans*, has a cell-autonomous inhibitory role in cell proliferation [5].

In a previous study we have shown that increasing DAF-16 activity by transgenic expression causes tumor-like germline hyperplasia that is accompanied by degradation of the extracellular matrix surrounding the gonad, and this eventually results in premature death [6]. Due to its similarity to several aspects of tumorigenesis in higher organisms, we suggest to term this phenotype a “*C*. *elegans* germline tumor”. The penetrance of this tumor-like phenotype is strongly enhanced in a loss-of-function mutant of *shc-1*, which encodes a DAF-2 interacting protein and homolog of the human adaptor protein Shc [6]. We had shown that DAF-16 activity exclusively in the hypodermis is sufficient to induce the tumor-like phenotype, arguing strongly in favor of a non-cell-autonomous mechanism. One of the surprising results of this study was that inactivation of *daf-2*, which should further increase DAF-16 activity according to the canonical IIS signaling pathway, rather suppresses DAF-16 mediated germline tumor phenotype. This suggests that the pro-oncogenic DAF-16 activity is positively rather than negatively regulated by IIS, providing a conundrum that could not be explained easily. Moreover, a loss-of-function mutation in *daf-18*/PTEN (Phosphatase and Tensin homolog), encoding a well-studied phosphatase with functions antagonistic to PI3K that, according to canonical insulin signaling, should enhance PI3K signaling and inactive DAF-16, further enhances this phenotype. Furthermore, the three AGC kinases AKT-1, AKT-2, and SGK-1 that in most previous studies have displayed redundant activities as negative regulators of DAF-16, have antagonistic roles in DAF-16 mediated tumor formation. AKT-1 acted as an inhibitor of germline tumor formation, as the *shc-1;akt-1* double mutant shows tumor-like growth similar to that observed in *shc-1;Is[daf-16*::*GFP]* [6]. Strikingly, down-regulation of *akt-2* or *sgk-1*, like *daf-2*, suppressed germline hyperplasia in *shc-1;akt-1* animals, indicating that the activity of DAF-2, AKT-2 or SGK-1 in this context contributed to germline tumor formation. All these observations are inconsistent with the canonical model of the linear IIS pathway, in which DAF-2 negatively regulates DAF-16. DAF-16 in turn has been shown before to inhibit germ cell proliferation cell-autonomously, and also acts to inhibit *gld-1* germline tumors [5, 7] that differ in certain aspects from the tumor-like phenotype observed here. These observations suggest a novel proliferation-promoting DAF-16/FOXO function in the presence of active IIS (a comparison of the novel *versus* canonical DAF-16 function and its regulation is summarized in the S1 Fig).

The *C*. *elegans* germline is the only tissue harboring stem cells and mitotic events throughout adulthood. The somatic distal tip cell (DTC) serves as the niche and maintains the germline stem cell fate by producing ligands to active Notch receptor on neighboring germ cells [8]. In addition, IIS promotes robust larval germline proliferation via inactivation of DAF-16 [5]. Moreover, the mammalian target of rapamycin complex one (mTORC1) signaling in the germline promotes cell proliferation and prevents differentiation [9]. Furthermore, the canonical TGF-ß/dauer signaling pathway has been shown to promote mitosis versus meiosis fate in parallel to Notch signaling [10]. Mutations causing hyperactive Notch signaling can lead to germline tumor due to excessive cell proliferation [11, 12]. In contrast, despite positive roles of IIS and mTORC1 signaling in regulating germline proliferation, active IIS contributing to germline tumor formation to our knowledge has only been reported once [6].

BMP signaling (also called the TGF-ß Sma/Mab pathway in *C*. *elegans*) is also highly conserved from invertebrates to humans. The BMP signaling cascade involves extracellular ligands like DBL-1, type I (SMA-6) and type II (DAF-4) receptors, R-Smads (SMA-2 and SMA-3), a co-Smad (SMA-4), and a transcription co-factor (SMA-9) [13–15]. BMP signaling in *C*. *elegans* positively regulates body size, male tail development and innate immunity [16–18]. In addition, reduction of BMP signaling and IIS delays reproductive aging by maintaining oocyte and germline quality through non-autonomous mechanisms [19].

Here, we uncover a novel interaction of BMP signaling and DAF-16 in promoting germline tumor formation. Our genetic and biochemical results suggest that the R-Smad proteins SMA-2 and SMA-3 bind directly to DAF-16 in the nucleus to regulate transcription of several common target genes of the mTORC1 pathway. R-Smad regulation of DAF-16 activity during germline tumorigenesis seems to be critical only in the hypodermis. Furthermore, we demonstrate that cell-autonomous and non-cell-autonomous activities of DAF-16 affect germline proliferation in opposite ways, suggesting that a functional balance of DAF-16 in distinct tissues may be important for rapid adaption to the diverse environmental change to ensure survival and reproduction.

## Results

### Both mTORC1 and BMP signaling pathways contribute to the DAF-16 dependent tumorous germline phenotype

**Fig 1:**
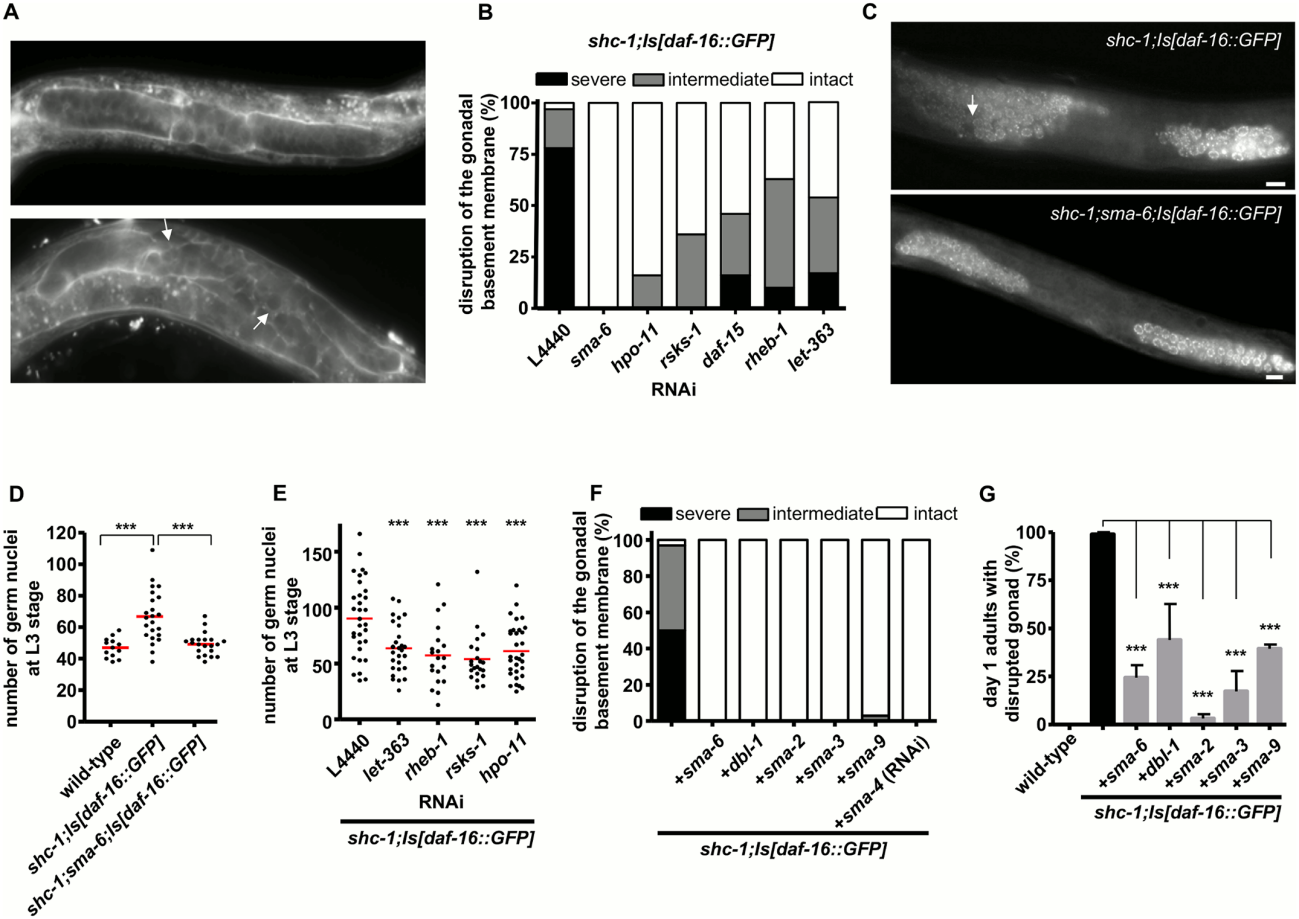
mTORC1 and BMP signaling pathways contribute to the DAF-16 dependent tumorous germline phenotype. (A) Representative pictures of MitoTracker stained gonadal basement membrane. The white arrows point to disrupted gonadal basement membrane. (B) RNAi knock-down of *sma-6*, *hpo-11* and the components of mTORC1 signaling suppresses the defects in the gonadal integrity in *shc-1;Is[daf-16*::*GFP]* animals. (C) Anti-PGL-1 germline antibody staining of *shc-1;Is[daf-16*::*GFP]* and *shc-1;sma-6;Is[daf-16*::*GFP]* L3 larvae. The white arrow points to hyperplasia in the anterior gonad arm of a representative *shc-1;Is[daf-16*::*GFP]* animal (top). In *sma-6* mutant background, hyperproliferation is suppressed (bottom). (D) *sma-6* mutation significantly decreases the elevated number of the germ cells in the anterior gonad arms of *shc-1;Is[daf-16*::*GFP]* L3 animals. Numbers of germ cells per gonad arm ± SEM: wild-type: 47 ± 2 (n = 13); *shc-1;Is[daf-16*::*GFP]*: 67 ± 4 (n = 23); *shc-1;sma-6;Is[daf-16*::*GFP]*: 49 ± 2 (n = 21) (P<0.0001 compared to *shc-1;Is[daf-16*::*GFP]* animals). (E) RNAi knock-down of the components of mTORC1 signaling and *hpo-11* significantly decreases the elevated number of the germ cells in *shc-1;Is[daf-16*::*GFP]* L3 animals. Numbers of germ cells per gonad arm ± SEM: L4440 control: 90 ± 6 (n = 32); *let-363* RNAi: 64 ± 4 (n = 28); *rheb-1* RNAi: 57 ± 6 (n = 20); *rsks-1* RNAi: 54 ± 5 (n = 22); *hpo-11* RNAi: 61 ± 4 (n = 32) (P<0.0001 compared to *shc-1;Is[daf-16*::*GFP]* + RNAi L4440 control animals). (F) Inactivation of genes in the BMP signaling pathway completely abolishes gonad disruption in *shc-1;Is[daf-16*:: *GFP]* L3 animals. (G) Inactivation of genes in the BMP signaling pathway strongly suppresses gonad disruption in *shc-1;Is[daf-16*:: *GFP]* day one adult animals. Scale bar: 10 μm. In this and the following figures, percentages of basement membrane defects are presented as mean ± SD; the mean values and statistical analysis are summarized in S2 Table; the asterisks represent: * P<0.01, ** P<0.001, and *** P<0.0001.

We had shown previously that both *shc-1* and *daf-16* which link JNK and IIS signaling pathways contribute to tumorous germline formation [20]. In order to dissect the contribution of each pathway, we performed an RNA interference screen to identify kinases involved in germline hyperplasia. For this purpose, we screened the contribution of all kinases in the Ahringer lab [21] and the ORFeome library [22]. As a readout, we looked for suppression of the 49% penetrant sterility of *shc-1;Is[daf-16*::*GFP]* animals, a phenotypic aspect that is the consequence of the tumorous germline (S2 Fig) [6]. We found that RNAi clones of three kinase genes, *sma-6*, *hpo-11*, and *rsks-1*, not only strongly suppressed sterility (S1 Table), but also germline hyperproliferation and rupture of the gonadal basement membrane (Fig 1A–1E, S3 Fig and S2 Table). This suggests that the activity of all three genes contributes to germline tumor formation in *shc-1;Is[daf-16*::*GFP]* mutant animals.

Among these identified three genes, *rsks-1* encodes the *C*. *elegans* S6 kinase homolog, a downstream target of mammalian target of rapamycin complex 1 (mTORC1) [23] which is activated by the GTPase RHEB-1/Rheb and the adapter protein DAF-15/Raptor [24–26]. This suggested that mTORC1 signaling might contribute to DAF-16 dependent germline tumor formation. We next asked whether inactivating other mTORC1 components in *C*. *elegans*, *let-363/*TOR, *rheb-1*/Rheb, and *daf-15*/Raptor, could also suppress the *shc-1;Is[daf-16*::*GFP]* tumor. RNAi knock-down of *let-363*, *daf-15* or *rheb-1*, respectively, resulted in constitutive L3 larval arrest in the F1 generation of *shc-1;Is[daf-16*::*GFP]* animals, substantiating previous findings that mTORC1 is necessary for normal development of *C*. *elegans* [27]. However, this prevented examination of the tumor phenotype that we examined at mid-late L3 larval stage [6]. Therefore, we initiated RNAi knock-down at larval stage L1 since such short-term RNAi knock-down of mTORC1 did not cause L3 larval arrest. RNAi knock-down of *daf-15*, *let-363* and *rheb-1*, respectively, strongly suppressed both germline hyperproliferation and disruption of the basement membrane of *shc-1;Is[daf-16*::*GFP]* mid-late L3 animals (Fig 1B and 1E). We conclude that mTORC1 in this genetic context also has oncogenic activity in *C*. *elegans*.

*sma-6* encodes the TGF-ß/BMP type I receptor [28], suggesting a role of BMP signaling in germline tumor formation. We asked whether the other genes in the BMP pathway are also involved the germline phenotype. Crossing *shc-1;Is[daf-16*::*GFP]* with *daf-4(m63)* strongly enhanced the low penetrance of the constitutive dauer phenotype of *daf-4*, and prevented examination of this strain in stages later than L3. Inactivating the BMP receptor ligand gene *dbl-1*, the Co-Smad gene *sma-4*, the Smad genes *sma-2*, *sma-3*, or the Smad cofactor gene *sma-9* by either mutation or RNAi fully suppressed the sterile phenotype of *shc-1;Is[daf-16*::*GFP]* and strongly decreased the percentage of animals with disrupted gonadal basement membrane in both L3 and day one adult animals (Fig 1F and 1G, S1 and S2 Tables). Therefore, in addition to mTORC1, also BMP signaling contributes to DAF-16 dependent germline tumor formation.

In *C*. *elegans*, a second TGF-ß/dauer signaling pathway regulates dauer formation [29, 30]. It had been suggested that neuronal TGF-ß dauer signaling promotes germline proliferation [10, 31]. We observed that RNAi knock-down of *daf-1* encoding the type I TGF-ß receptor (specifically involved in dauer signaling) only slightly suppressed sterility, but failed to decrease the severity of the gonadal defect in *shc-1;Is[daf-16*::*GFP]* animals (S2 Table). Therefore, *daf-1* RNAi behaved differently from *sma-6* RNAi, suggesting that TGF-ß/dauer signaling probably affects the DAF-16 mediated germline phenotype only indirectly.

### The interaction of DAF-16 and BMP signaling is specific to germline signaling

**Fig 2:**
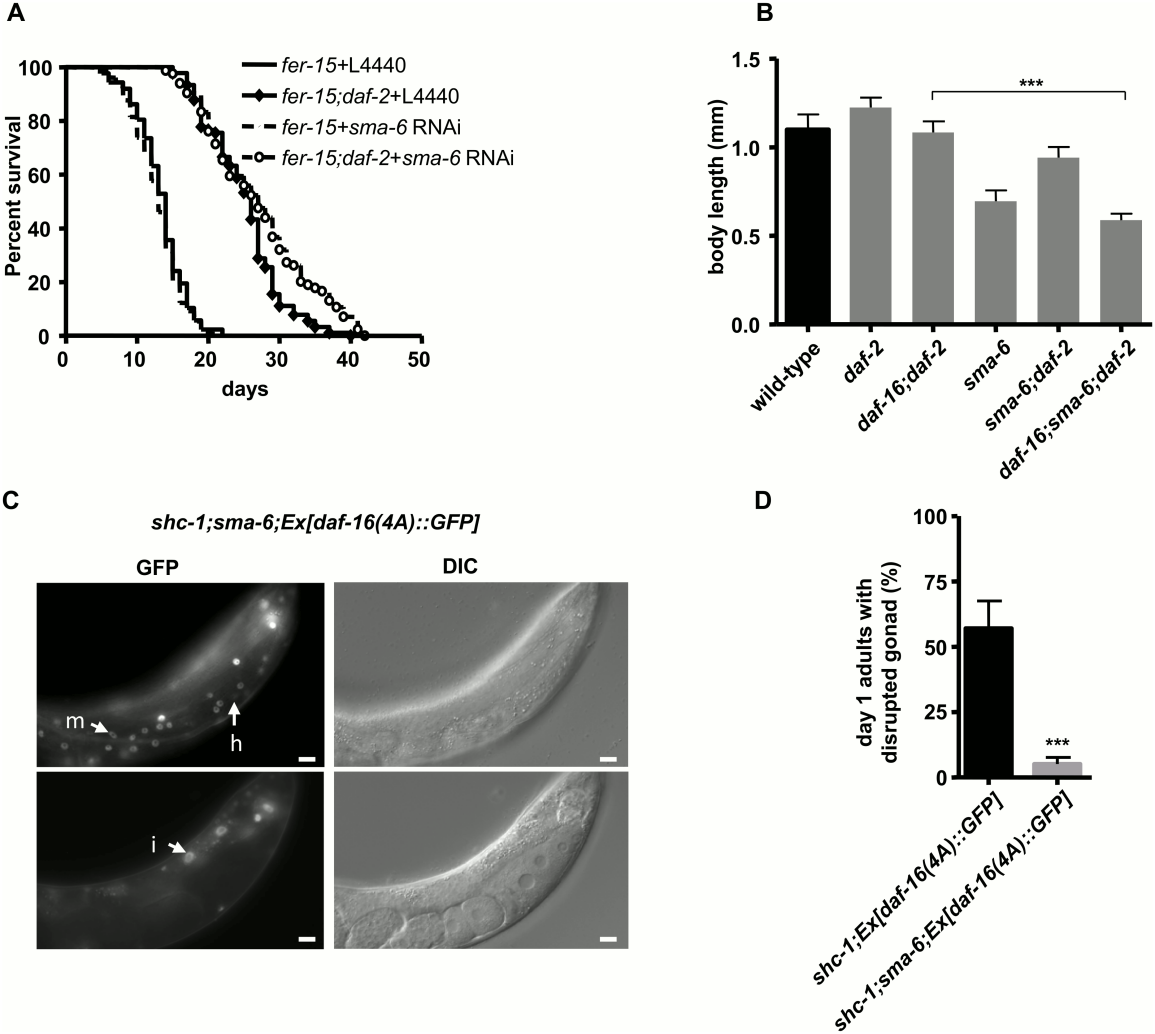
DAF-16 and BMP signaling do not cooperate in regulation of lifespan or body size. (A) Lifespan extension resulting from DAF-16 activation upon reduced IIS does not require SMA-6. Since *sma-6* mutant animals have a strong egg-laying defect, lifespan was performed in a temperature sensitive *fer-15* mutant background, in which animals become sterile at 25°C due to defective sperm formation [33]. Mean lifespan and statistical analysis are summarized in the S3 Table. (B) DAF-16 and BMP signaling regulate body size independently. Average body length are presented as mean ± SD. N2: 1.10 ± 0.09 mm (n = 17); *daf-2(e1370)*: 1.22 ± 0.06 mm (n = 14); *daf-16(mu86);daf-2(e1370)*: 1.08 ± 0.06 mm (n = 19); *sma-6(wk7)*: 0.70 ± 0.06 mm (n = 15); *sma-6(wk7);daf-2(e1370)*: 0.94 ± 0.07 mm (n = 10); *daf-16(mu86);sma-6(wk7);daf-2(e1370)*: 0.59 ± 0.04 mm (n = 21). (C) *sma-6* mutation does not affect nuclear localization of DAF-16(4A)::GFP in *shc-1;Ex[daf-16(4A)*::*GFP]* animals. Arrows: DAF-16::GFP in the hypodermal (h), the muscle (m) and intestinal nuclei (i). (D) *sma-6* mutation strongly suppresses the gonadal basement membrane defect in *shc-1;Ex[daf-16(4A)*::*GFP]* animals.

Since we discovered, to our knowledge, a novel functional cross-talk between BMP signaling and DAF-16, we asked whether they also interact to promote additional functions of each other. Active DAF-16 under reduced IIS leads to constitutive dauer formation at larval stage and lifespan extension during adulthood [32]. Therefore, we next tested whether BMP signaling is also required for DAF-16 to regulate lifespan extension or dauer formation. *sma-6* RNAi knock-down did not shorten, but rather slightly extend the lifespan of *daf-2(e1370)* animals (Fig 2A and S3 Table). *sma-6(wk7)* also did not suppress constitutive dauer formation of *daf-2(e1370)* at 25°C (S4 Table). These data suggest that SMA-6/BMP function does not seem to be involved in other roles of DAF-16.

One of the best studied functions of BMP signaling in *C*. *elegans* is in body size regulation [28]. IIS also regulates body size, since *daf-2* mutant animals are longer than wild type as a consequence of DAF-16 activation [34], results that we confirmed here (Fig 2B and S5 Table). However, *sma-6(wk7)* reduced body sizes of both *daf-2* and *daf-16;daf-2* animals, suggesting that BMP signaling positively regulates body size probably in parallel to DAF-16. Taken together, signaling to the germline is the only cooperative role of BMP and DAF-16 interaction that we found.

### BMP signaling promotes the stem-cell proliferative activity of nuclear DAF-16

DAF-16 activity is regulated at least at two steps: via control of its nuclear translocation and, subsequently, via modifying its strength as a transcriptional activator through protein interactions and possibly protein modifications. The DAF-16(4A) variant shows constitutive nuclear localization and our previous study has indicated that both DAF-16(4A)::GFP and wild type DAF-16::GFP expressed in *shc-1(ok198)* background induce germline tumorigenesis [6, 35]. Nuclear localization of DAF-16(4A)::GFP was not altered by a *sma-6* mutation (Fig 2C). However, *sma-6(wk7)* strongly reduced the penetrance of the germline phenotype in *shc-1;Ex[daf-16(4A)*::*GFP]* animals (Fig 2D and S2 Table). This suggests that SMA-6 may modulate the transcriptional activity of nuclear DAF-16.

### BMP signaling and DAF-16 promote the germline hyperproliferation in a non-cell-autonomous way

**Fig 3:**
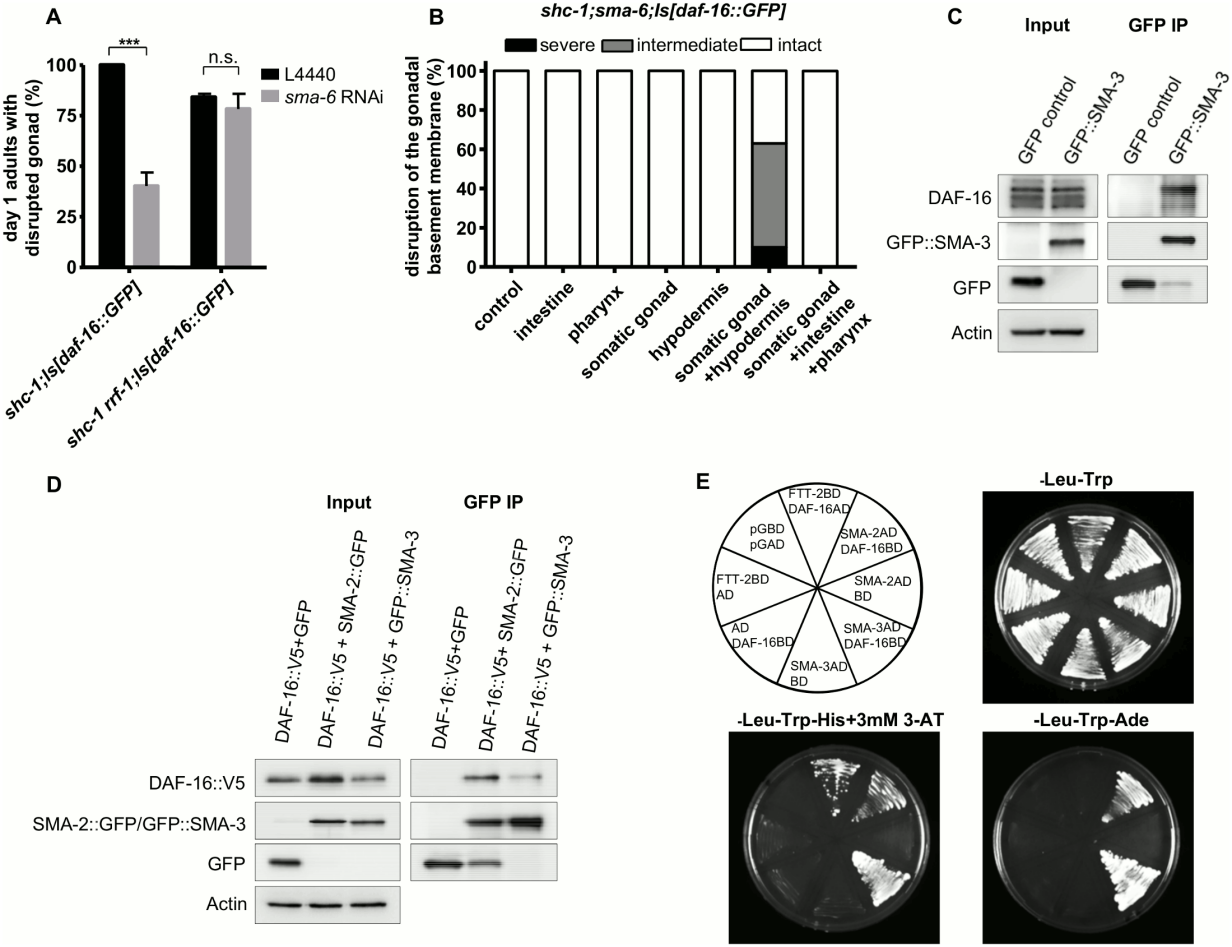
BMP signaling pathway interacts with DAF-16 in the hypodermis. (A) *sma-6* RNAi in the germline does not suppress the tumorous germline phenotype in *shc-1;Is[daf-16*::*GFP]* animals. (B) Only combined expression of *sma-6* in the hypodermis and the somatic gonad of *shc-1;sma-6;Is[daf-16*::*GFP]* animals rescues the *sma-6(wk7)* suppression of DAF-16 germline tumor phenotype, as indicated by disruptions of the gonadal basement membrane. F1 transgenic animals were analyzed for the hypodermis-specific rescue by *sma-6* in *shc-1;sma-6;Is[daf-16*::*GFP]*, since we failed to get stable transgenic lines in *shc-1;sma-6;Is[daf-16*::*GFP]* background but could receive several transgenic line in *sma-6* single mutant background, by using the hypodermis-specific promoter. All the transgenic lines are summarized in the S6 Table. (C) DAF-16 coimmunoprecipitates with GFP::SMA-3. Sonicates were prepared from mixed stages of *GFP*::*sma-3* transgenic animals. An *ife-2* GFP transcriptional fusion reporter was used as GFP negative control. All samples contained 5 mg of total protein. (D) DAF-16 binds to both SMA-2 and SMA-3 in yeast. Top left: The arrangement of the tested interactions; -Leu-Trp: transformation control of the constructs in yeast; -Leu-Trp-His+3mM 3-AT: Histidine selection revealed interactions between SMA-3/DAF-16, SMA-2/DAF-16 and FTT-2/DAF-16 (3 mM 3-AT); -Leu-Trp-Ade: Adenine selection revealed interactions of SMA-3/DAF-16 and SMA-2/DAF-16. (E) V5 tagged DAF-16 coimmunoprecipitates with both GFP tagged SMA-2 and SMA-3 in HEK293T cells.

Reporter gene studies have suggested that *sma-6* is expressed in the hypodermis, intestine, and pharynx [28]. Using our own reporter genes, we also observed weak *sma-3* expression in the somatic gonad (S4 Fig). It is not known whether *sma-6* is expressed in the germline due to germline silencing of the transgenic reporter genes used in such expression analysis. To knock-down *sma-6* exclusively in the germline, we performed *sma-6* RNAi in the background of the *rrf-1(ok589)* loss-of-function mutant that limits the sensitivity against RNA interference to the germline [36]. *sma-6* RNAi suppressed the gonadal integrity defects in *shc-1;Is[daf-16*::*GFP]*, but not in *shc-1;rrf-1;Is[daf-16*::*GFP]* animals (Fig 3A, S1 and S2 Tables), indicating that somatic, but not germline activity of SMA-6 may contribute to DAF-16 dependent tumors formation. Since a recent report suggested that *rrf-1(ok589)* to some extent also might allow RNAi to work in the intestine and seam cells [37], we cautiously argue that neither seam cell nor intestinal SMA-6 activity seem to contribute to the DAF-16 dependent germline tumor phenotype.

In order to pinpoint the critical tissue for BMP activity, we expressed *sma-6* under the control of tissue specific promoters in *shc-1;sma-6;Is[daf-16*::*GFP]* animals to rescue tumor suppression of *sma-6(wk7)* (rescue should result in reoccurrence of the tumor phenotype). Neither expression of *sma-6* in the intestine, pharynx nor in the somatic gonad resulted in the reappearance of germline tumors in *shc-1;sma-6;Is[daf-16*::*GFP]* animals (Fig 3B). We tried in vain to receive stable transgenic lines of *Pdpy-7*:*sma-6* in *shc-1;sma-6;Is[daf-16*::*GFP]* background, in which *sma-6* should be expressed exclusively in the hypodermis. Although we obtained more than 200 F1 transgenic animals, we never observed *sma-6* expression in the F2 generation, indicating either toxicity or silencing of this transgene. Therefore, we analyzed F1 transgenic animals with hypodermal-only *sma-6* expression, but these did not show rescue. However, coexpression of *sma-6* in both hypodermis and somatic gonad was sufficient to rescue *sma-6* tumor suppression. In contrast, expressing *sma-6* in all *sma-6* positive tissues except the hypodermis (somatic gonad, intestine and pharynx), did not cause tumor reoccurrence. We conclude that the hypodermis and somatic gonad are the critical tissues for BMP signaling that promote DAF-16 tumor induction.

### SMA-2 and SMA-3 bind directly to DAF-16

Since it has been shown that forkhead transcription factors can bind to Smad proteins directly [38, 39], a simple hypothesis was that DAF-16 might physically interact with R-Smad proteins SMA-2 and/or SMA-3 in the hypodermis. We tested R-Smad and DAF-16 interactions with three different assays.

First, we performed *in vivo* co-immunoprecipitation experiments from *C*. *elegans* cell extracts. Extracts from animals carrying functional *GFP*::*sma-3* were precipitated with anti-GFP antibody and the resulting precipitates were subjected to Western blotting by using anti-DAF-16 antibody to detect endogenous DAF-16 (Fig 3C). We detected DAF-16 in GFP::SMA-3 but not in GFP precipitates, suggesting a specific interaction between DAF-16 and SMA-3. We were unable to express a functional version of tagged SMA-2 in *C*. *elegans*, since all fusion tags (GFP, Flag and V5 tags) we had tested resulted in loss of SMA-2 function, indicated by a failure of the respective transgene to rescue the small body size phenotype of *sma-2(e502)* animals.

Second, we performed IP with recombinant DAF-16::V5 and SMA-2::GFP/GFP::SMA-3 co-expressed in the human HEK293T cell line to verify the physical interaction. DAF-16::V5 protein was coimmunoprecipitated with both SMA-2::GFP and GFP::SMA-3 (Fig 3D), further supporting a direct interaction between the two R-Smad proteins and DAF-16.

Third, we tested whether DAF-16/R-Smad interactions occur in a yeast model. As positive control we included FTT-2, which is a *C*. *elegans* 14-3-3 homologue and has been shown to interact with DAF-16 [40]. We found that in a yeast-two-hybrid system, FTT-2, SMA-2, and SMA-3 all interact with DAF-16 (Fig 3E), corroborating our co-immunoprecipitation results. Even though it has been suggested previously that SMA-2 and SMA-3 form a heterodimer [41], we were unable to detect a direct interaction between SMA-2 and SMA-3 in this assay (S5 Fig).

### DAF-16 and BMP signaling share common target genes

**Fig 4:**
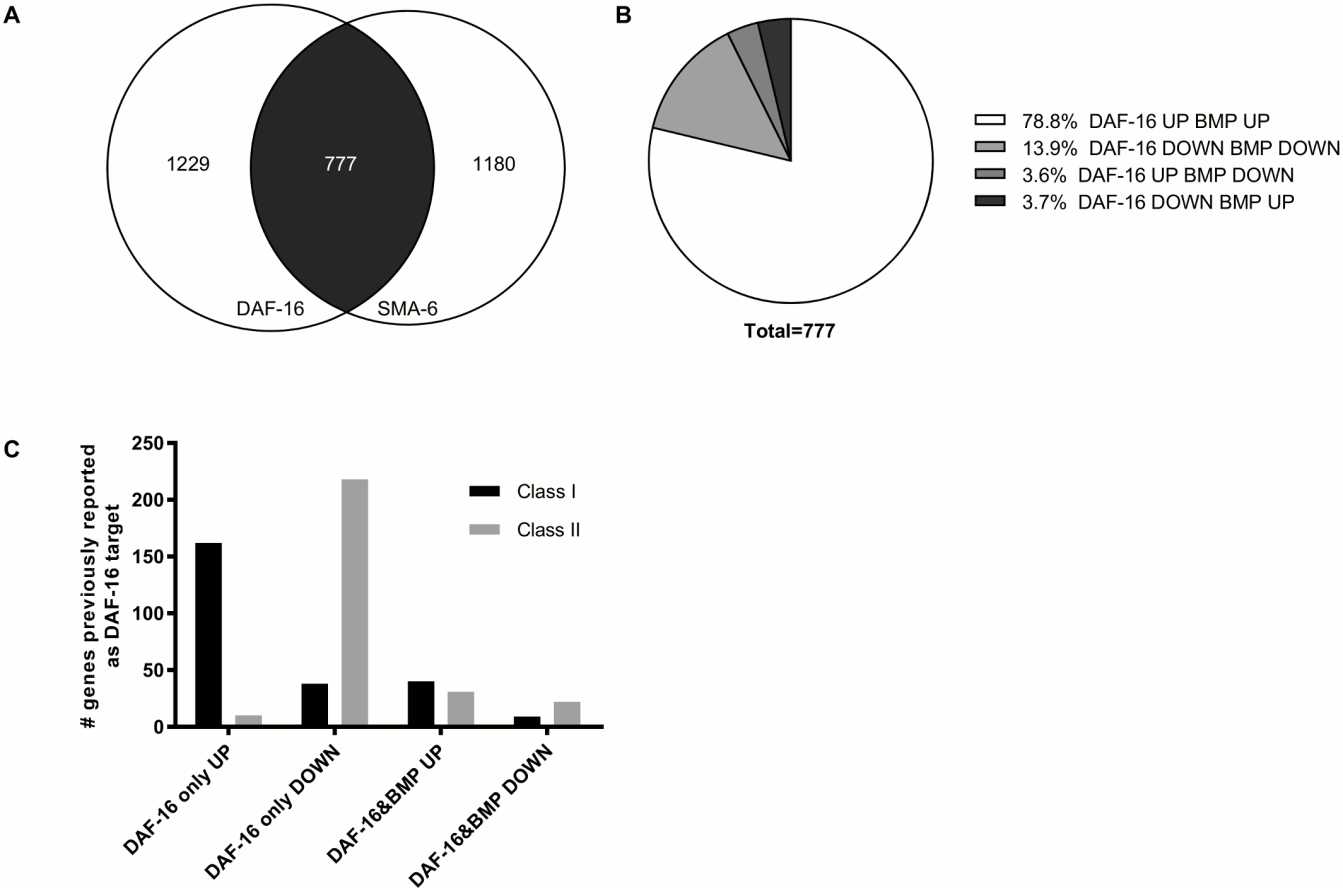
Comparison of expression of DAF-16 and BMP signaling regulated genes. (A) Overlap in the transcriptional outputs of DAF-16 and BMP signaling. 2,006 genes are significantly regulated by DAF-16 by comparing *shc-1(ok198);Is[daf-16*::*GFP]* and *shc-1(ok198) daf-16(mu86)* animals; 1,975 genes respond significantly to SMA-6 by comparing *shc-1(ok198);Is[daf-16*::*GFP]* and *shc-1(ok198);sma-6(wk7);Is[daf-16*::*GFP]*. 777 genes among them are regulated by both DAF-16 and SMA-6. (B) Classification of the response patterns of 777 genes responding to both DAF-16 and BMP signaling. Most genes are either upregulated or downregulated in response to both factors; opposing transcriptional effects were observed for only 57 genes. (C) Genes responding to DAF-16 in a SMA-6 independent manner are more strongly enriched in the known class I and II DAF-16 targets than genes responding to both DAF-16 and SMA-6.

Since both DAF-16 and its BMP interactors SMA-2 and SMA-3 encode transcription factors, we next sought to determine downstream genes influenced by both sets of proteins. Several reports have already documented candidate genes directly regulated by DAF-16 or BMP signaling, but genes co-regulated by DAF-16 and SMA-2/3 have not been reported so far. We therefore performed microarray assays to identify genes differentially regulated in *shc-1(ok198);Is[daf-16*::*GFP]*, *shc-1(ok198) daf-16(mu86)* and *shc-1(ok198);sma-6(wk7);Is[daf-16*::*GFP]* strains. Hierarchical clustering of genes differentially regulated between any of the three strains suggested a substantial effect of BMP signaling on the transcriptional output of DAF-16 (S6 Fig). To analyze the consequences of this interaction in more detail, we used a linear model analysis to select genes with a statistically significant (FDR-corrected p-value < 0.01) and robust (at least two-fold expression change) response to DAF-16 or SMA-6 activity changes. We identified 2,006 genes as regulated by DAF-16 and 1,957 genes as regulated by SMA-6. Intriguingly, 777 of these genes respond to both DAF-16 and SMA-6, which corresponds to 39% and 40% of all genes regulated by DAF-16 and by SMA-6, respectively (Fig 4A). A more detailed investigation of these genes revealed that 79% (612/777) of the genes that at least partially depend on SMA-6 are genes that are activated by DAF-16, and another 108 genes are downregulated by DAF-16 in a SMA-6-dependent manner (Fig 4B). Together this indicates that 94% of the genes responsive to both factors are controlled by them in a cooperative manner.

**Fig 5:**
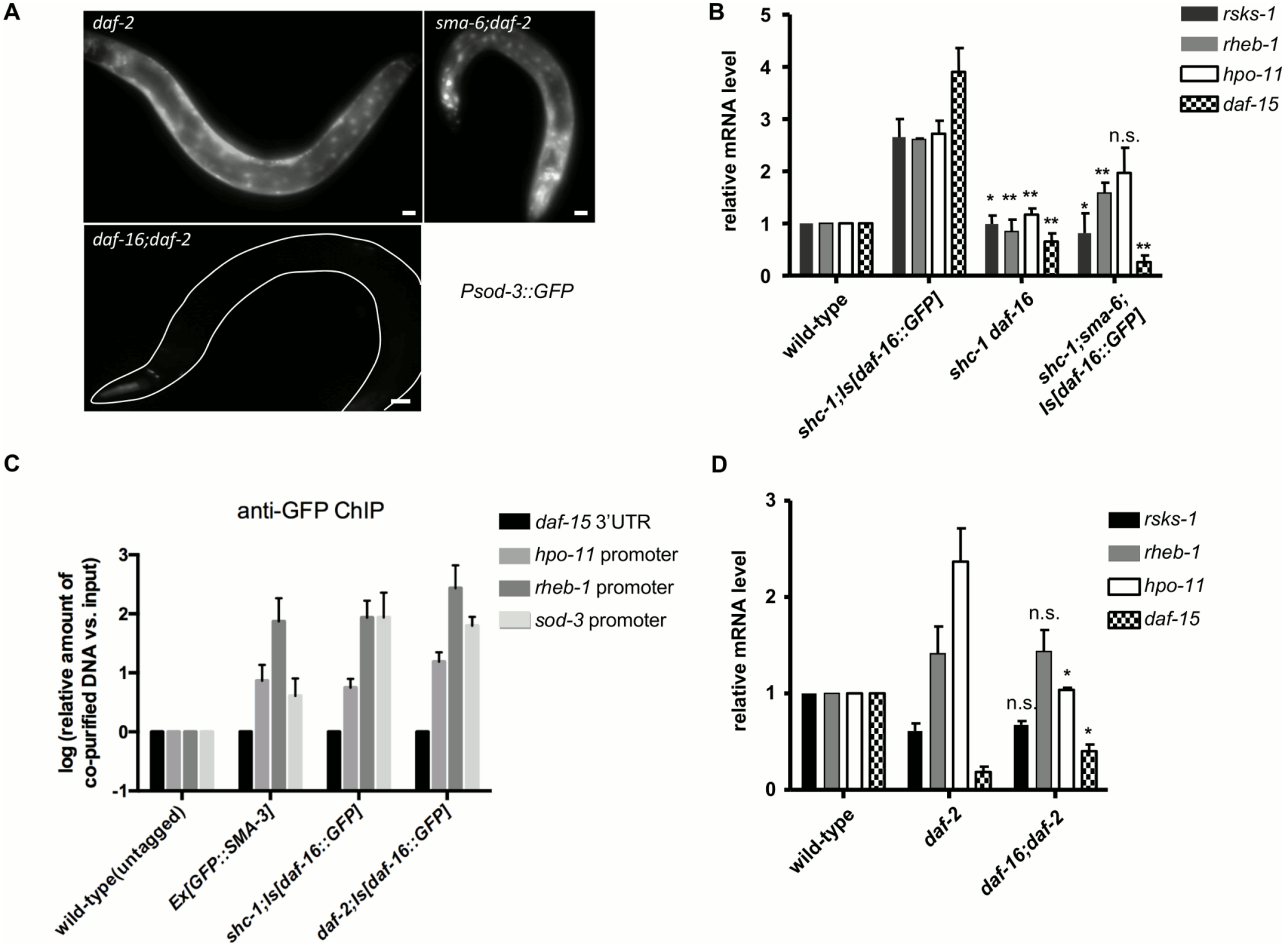
Transcriptional activation of TORC1 and HPO-11 by BMP signaling and DAF-16. (A) Expression of *Psod-3*::*GFP* in *daf-2* animals is DAF-16, but not SMA-6 dependent. (B) Transcription of candidate target genes of DAF-16 and SMA-2/3 are activated in *shc-1;Is[daf-16*::*GFP]* animals. Mutation in either *daf-16* or *sma-6* suppresses their activation. P-values are summarized in the S8 Table. (C) DAF-16 and SMA-3 binding is enriched in the promoter regions of *hpo-11* and *rheb-1* genes. Enrichment was determined by ChIP using α-GFP antibody for DAF- 16::GFP or GFP::SMA-3 and normalized to *daf-15* 3’UTR region that is supposed not to be bound by DAF-16 or SMA-3. Error bars represent the SEM. (D) Transcriptional regulation of the four candidate target genes by DAF-16 in *daf-2* mutant animals. P-values are summarized in the S8 Table.

Next we compared the genes identified in our analysis with previously reported consensus sets of genes that are activated (class I genes) and repressed (class II genes) by DAF-16 and that were derived from the combined analysis of different genome-wide expression studies. Typically, these compared *daf-2(-)* against *daf-16(-);daf-2(-)* backgrounds [42]. We found that 33% (162 of 498) of the genes we identify as upregulated by DAF-16 in a SMA-6 independent manner are known class I target genes. Similarly, 30% (218 of 731) of the genes that we see downregulated by DAF-16 independent of SMA-6 are known class II target genes. In contrast, only 8.6% (62 of 720) of the genes either up- or downregulated cooperatively by DAF-16 and SMA-6 are known class I or class II target genes, respectively (Fig 4C). This suggests that the common targets of DAF-16 and BMP signaling are typically distinct from the DAF-16 targets that were identified by comparing *daf-2(-)* and *daf-16(-); daf-2(-)* gene expression. To confirm this hypothesis, we also checked whether modulation of BMP signaling could alter expression of the frequently used DAF-16 target gene *sod-3*. In *daf-2(e1370)* mutant, *sod-3* expression is increased in a DAF-16 dependent manner and this can be visualized *in vivo* by using an integrated transcriptional GFP fusion reporter [43]. We verified this observation and showed that *daf-2* animals showed increased GFP expression, and this was fully dependent on DAF-16 (Fig 5A). Knock-down of *sma-6* did not significantly affect the expression level of the *sod-3*::*GFP* reporter in *daf-2* background, further supporting our microarray assay results.

### TORC1 and *hpo-11* are common target genes of DAF-16 and BMP signaling to promote germline tumor

Next, we searched for common targets of DAF-16 and BMP signaling to cause germline tumor formation. Inspecting the modENCODE database, we found reports of DAF-16 occupancy on the promoter regions of *hpo-11*, *daf-15*, *rsks-1* and *rheb-1* (S7 Fig). In addition, we identified consensus DAF-16 binding sites in each of their 5’ promoter sequences, making them candidates for being direct DAF-16 target genes. Next, we tested by mRNA quantification whether expression of these genes is affected by DAF-16 and BMP activities. Quantitative-PCR (q-PCR) results showed that the mRNA levels of *rsks-1*, *rheb-1*, *daf-15*, and *hpo-11* in *shc-1;Is[daf-16*::*GFP]* animals were strongly increased when compared to wild-type, and the increase fully depended on DAF-16 (Fig 5B). Inactivation of *sma-6* significantly reduced mRNA levels of *rsks-1*, *rheb-1*, *daf-15*, but not of *hpo-11*. In summary, combinatorial activation by DAF-16 and R-Smad transcription factors in the hypodermis may activate some common transcriptional target genes to cause tumor formation in the germline.

To show direct binding of DAF-16 and SMA-3 to the promoters of the candidate target genes, we performed ChIP-qPCR experiments using the promoter regions predicted by the modENCODE project (position of primer sets for ChIP-qPCR indicated in the S7 Fig). Our data revealed binding of both DAF-16 and SMA-3 in the *rheb-1* promoter, and binding of DAF-16 in the *hpo-11* promoter (Fig 5C). Quantification of DAF-16 and SMA-3 binding on both *daf-15* and *rsks-1* promoters failed, since we were unable to find primer sets suitable for q-PCR quantification (list of tested primer sets is provided in S9 Table).

### IIS controls transcriptional specificity of DAF-16 towards its target genes

One of the surprising results in Qi et al. (2012) was that DAF-16 dependent germline tumor formation was suppressed, rather than enhanced by additional inactivation of *daf-2*. This indicated an obvious discrepancy to the canonical model of the IIS pathway, which would predict that down-regulation of *daf-2* should enhance, rather than suppress DAF-16 activities. We asked whether inactivation of *daf-2* suppresses DAF-16 dependent tumor phenotype via regulating the identified downstream genes. Whereas all four candidate genes (*rsks-1*, *rheb-1*, *daf-15*, and *hpo-11)* are strongly activated in the tumor strain *shc-1;Is[daf-16*::*GFP]* that carries a *daf-2(+)* allele, they were differentially regulated in *daf-2(-)* animals (Fig 5D). In *daf-2* mutant animals only *hpo-11* mRNA level was up-regulated by DAF-16. In contrast, both *rsks-1* and *daf-15* transcripts were strongly reduced and only down-regulation of *daf-15* mRNA level partially depended on DAF-16. This suggests the existence of both DAF-16 dependent and independent mechanisms to inactivate mTORC1 in *daf-2(-)* mutant background. In addition, our results indicate that the target specificity of DAF-16 can be differentially affected by upstream IIS.

### Hypodermal DAF-16 and BMP signaling promote germline proliferation

**Fig 6:**
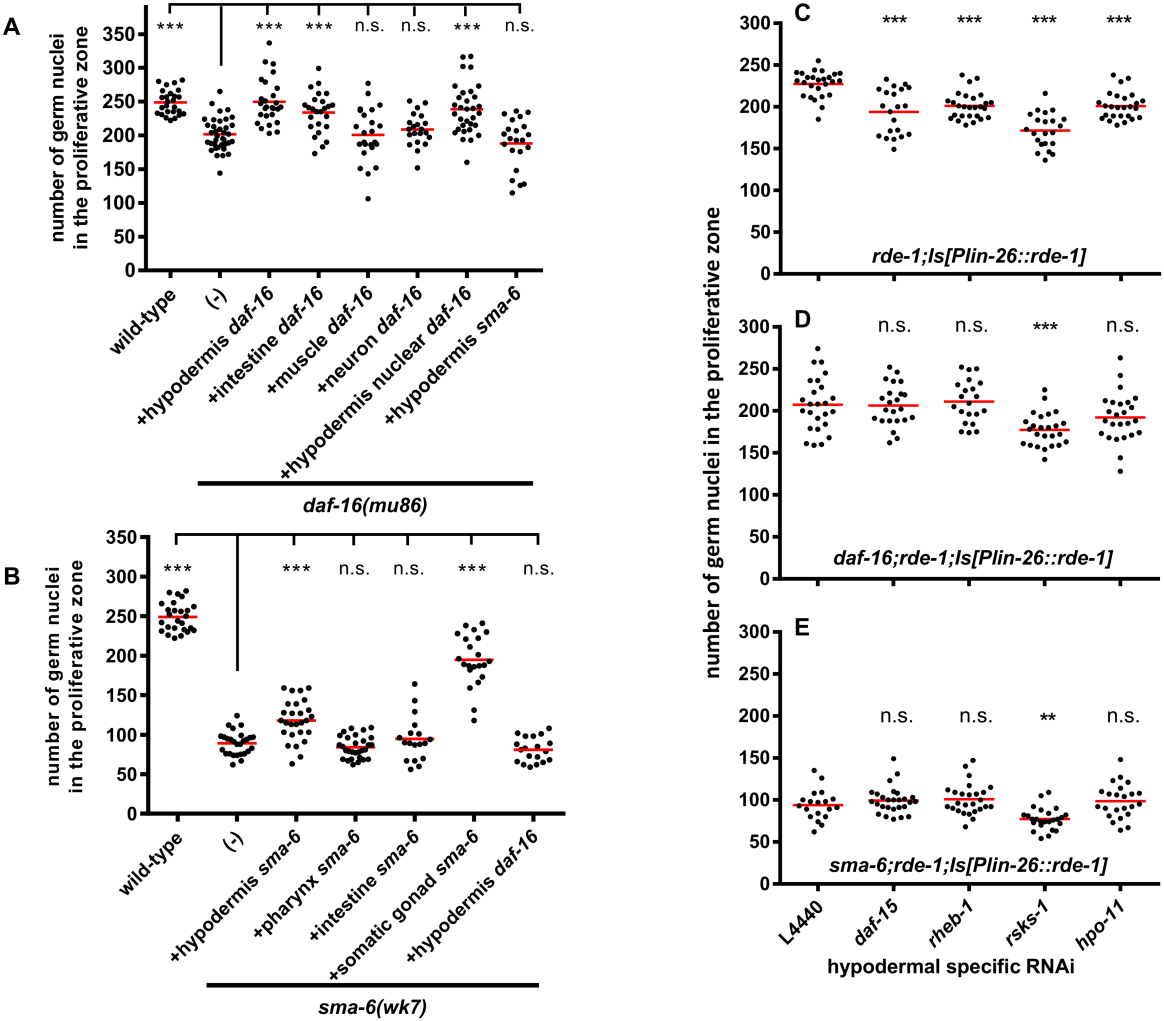
DAF-16 and BMP signaling positively regulate germline proliferation. (A) DAF-16 promotes germline proliferation in a non-cell-autonomous way. (B) BMP signaling promotes germline proliferation in a non-cell-autonomous way. (C) Hypodermis-specific RNAi against *rsks-1*, *rheb-1*, *daf-15* and *hpo-11* reduces mitotic germ cell numbers. (D) Hypodermis-specific RNAi against *rsks-1*, *rheb-1*, *daf-15* and *hpo-11* in *daf-16* mutant animals. (E) Hypodermis-specific RNAi against *rsks-1*, *rheb-1*, *daf-15* and *hpo-11* in *sma-6* mutant animals. The average numbers of germ cell nuclei in the proliferation zone of day one adult animals ± SEM and P-values are summarized in the S9 Table.

The results so far suggest that DAF-16 mis-regulation as a consequence of distinct genetic backgrounds requires BMP signaling to become tumorigenic. The obvious question therefore was, whether BMP signaling also contributes to normal developmental functions of DAF-16 in wild-type strains, such as controlling germline proliferation. This could be examined by counting germline nuclei in the proliferative zone of the germline which is located between the DTC and the transition zone in which germ cell differentiation is initiated. *daf-16* and *sma-6* animals at day one of adulthood contained approximately 20% and 63% less mitotic germline nuclei, respectively, than wild-type animals (Fig 6A), suggesting that both DAF-16 and SMA-6 positively regulate distal germline proliferation. To address tissue specificity of DAF-16 and SMA-6, we tried rescuing *daf-16* and *sma-6* in a tissue-specific way. Transgenic expression of both wild-type DAF-16::GFP and constitutive nuclear DAF-16(4A)::GFP in the hypodermis completely rescued the decreased number of mitotic germline nuclei in *daf-16* mutant animals. DAF-16 expression in the intestine also partially rescued the germline proliferation phenotype of *daf-16* mutants whereas expression in the neuron or muscle had no effect. We noticed that, whereas in the majority of animals hypodermal DAF-16 expression of either wild-type *daf-16*::*GFP* or *daf-16(4A)*::*GFP* rescued the *daf-16* mutant phenotype, in approximately 10% of these animals the numbers of mitotic germline nuclei even exceeded 300, something never observed in wild type animals. This sometimes led to enlargement of distal gonads (S8 Fig). Expression *sma-6* in the somatic gonad robustly rescued germline proliferation defect while hypodermis expression *sma-6* showed a minor, but significant, increase of proliferative germ cell number (Fig 6B). *sma-6* expression in neither intestine nor pharynx had any effect on *sma-6(-)* animals. Taken together, these results suggest both hypodermal DAF-16 and BMP signaling are required for wild-type germline proliferation. And they also act in additional distinct tissues.

Do DAF-16 and BMP functions in the hypodermis depend on each other? Hypodermal expression of *sma-6* failed to increase germ nuclei number in the proliferative zone of *daf-16* animals (Fig 6A), and hypodermal *daf-16* expression also did not increase the number of proliferative germ cells in a *sma-6* mutant (Fig 6B), These results suggest that the DAF-16 and BMP signaling activities in the hypodermis to regulate germline proliferation depend on each other, consistent with our proposed model that DAF-16 and R-Smad protein interaction cooperate to control common target genes.

Next we asked whether selective down-regulation of the target genes of DAF-16/SMA-2/3 interaction in the hypodermis, namely *rsks-1*, *rheb-1*, *daf-15* but also *hpo-11*, affects germ cell proliferations. For this purpose, we performed hypodermis-specific RNAi knock-downs of these four genes in an *rde-1;Is[Plin-26*::*rde-1]* strain. This strain harbors the argonaute/PIWI gene *rde-1* required for the function of RNA interference exclusively in the hypodermis. *rheb-1*, *daf-15*, or *hpo-11* RNAi resulted in an approximately 20% reduction of germline nuclei in the proliferative zone and *rsks-1* RNAi led to an approximately 25% reduction (Fig 6C), indicating that RHEB-1, RSKS-1, DAF-15, and HPO-11 in the hypodermis positively regulate germline proliferation. We asked whether hypodermal specific RNAi knock-downs of these four genes could further reduce number of proliferative germ nuclei in either *daf-16* or *sma-6* background. *rheb-1*, *daf-15*, or *hpo-11* RNAi failed to further reduce number of proliferative germ nuclei in either *daf-16;rde-1;Is[Plin-26*::*rde-1]* or *sma-6;rde-1;Is[Plin-26*::*rde-1]* animals (Fig 6D and 6E). *rsks-1* RNAi further reduced number of proliferative germ nuclei in these two strains but to less extent compared with *rde-1;Is[Plin-26*::*rde-1]* (15% *vs*. 25% reduction). Taken together, these experiments indicate that *rsks-1*, *rheb-1*, *daf-15* and *hpo-11* are epistatic to both DAF-16 and BMP signaling in the hypodermis to promote germline proliferation. In addition, *rsks-1* might also mediate additional pathway to regulate cell proliferation.

## Discussion

We identified protein interactions of DAF-1/FOXO and the Smad transcription factors of TGF-ß/BMP signaling that regulate target genes components of the mTORC1 pathway in *C*. *elegans*. This cross-talk promotes cell proliferation of germline stem cells non-cell-autonomously, and its hyper-activation results in a tumor-like germline phenotype. Another identified downstream target of IIS/DAF-16 axis encodes HPO-11, a pseudo-kinase with unknown function in *C*. *elegans* and ortholog of NRBP/MADM (nuclear receptor-binding protein, myeloid-leukemia factor-adaptor molecule), a regulator of germline stem cells and tumorigenesis in humans and *Drosophila*. Based on these functional similarities, we suggest that aspects of FOXO/R-Smad signaling to the germline stem cells may be conserved in evolution.

### BMP signaling contributes to DAF-16 dependent germline tumor formation

DAF-16/FOXO hyperactivity in the hypodermis results in a tumor-like germline phenotype that we showed to involve germ cell hyper-proliferation and disruption of the basement membrane [6]. Now we identified genes whose down-regulation suppressed this phenotype, among them *sma-2*, *sma-3*, *sma-4*, *sma-6* and *sma-9*, encoding components of the TGF-ß/BMP pathway. A combination of genetic and biochemical experiments furthermore revealed that the BMP effectors SMA-2 and SMA-3 can physically interact with DAF-16 to promote germline proliferation at wild type conditions, but may cooperate with hyper-active DAF-16 to cause tumors. This interaction is selectively required for only a subset of DAF-16 functions, based on the following arguments: (I) We show that the well-known beneficial roles of DAF-16 in extending lifespan and in promoting dauer formation in *daf-2(-)* animals do not involve BMP signaling (Fig 2, S3 and S4 Tables). (II) The DAF-16 dependent transcriptional activation of *sod-3* in *daf-2* mutant animals is also independent of BMP (Fig 5A). (III) Moreover, both BMP and IIS/DAF-16 contribute to the regulation of body size, but do so independently of one another (Fig 2C and S5 Table). Taking together, these results suggest that the functional interaction between BMP signaling and DAF-16 has consequences that seem to be specific for the germline, even though this interaction takes place in the hypodermis, a tissue adjacent to the gonad. Although both DAF-16 and R-Smad proteins are capable of shuttling between nucleus and cytoplasm, we suggest that the relevant interaction described here occurs in the nucleus, since a *sma-6* mutation could still suppress the phenotype caused by constitutively nuclear DAF-16(4A)::GFP. We therefore propose that BMP signaling promotes the activity of nuclear DAF-16, rather than its nuclear entry (Fig 2C and 2D), by modulating DAF-16 binding to the promoters of downstream genes. This claim was further justified by identifying DAF-16 and SMA-3 binding sites in the promoter of *rheb-1* gene of the mTORC1 pathway (see below).

Hypodermal activity of BMP signaling to the germline has been described to regulate oocyte and germline quality maintenance, but this was suggested to occur independently of IIS [19]. Our newly discovered functional cross-talk between DAF-16 and BMP is distinct of the previously identified interactions of DAF-16 with the canonical TGF-ß/dauer signaling pathway involved in lifespan regulation [44]. TGF-ß/dauer signaling in the nervous system also promotes germline proliferation via preventing meiosis [10, 31]. Even though we observed that down-regulating the *daf-1* gene, encoding a receptor type I homologue of the TGF-ß/dauer, but not the BMP signaling pathway, slightly decreased the severity of the DAF-16 dependent germline tumor phenotype, we consider the interaction between DAF-16 and canonical TGF-ß/dauer pathway most likely as being indirect, since DAF-16 and DAF-1 act in different tissues [6, 10].

### BMP signaling and DAF-16 control common targets to cause tumorous germline phenotype

We show that the R-Smad/DAF-16 interaction activates the expression of at least three genes (*rsks-1*, *rheb-1*, and *daf-15)* which encode components of the mTORC1 pathway. Down-regulation of each of them strongly suppressed tumorigenesis. In addition, DAF-16 activates *hpo-11* possibly independent of BMP signaling, although the influence of *hpo-11* on wild type mitotic germ cell numbers (Fig 6C) is fully lost in both *daf-16(mu86)* and *sma-6(wk7*) mutant backgrounds (Fig 6D and 6E). All four down-stream genes contain DAF-16 binding elements (DBE) or DAF-16 associate elements (DAE) in their 5’ promoter regions and *hpo-11* has been reported to be a DAF-16 direct target [45]. Even though arguments were convincing that DAE may be a recognition site of PQM-1, the transcription factor acting antagonistically to DAF-16 [42], the *C*. *elegans* modENCODE project detected direct binding of DAF-16 onto the promoter regions of *rheb-1* that contains DAE, rather than DBE [46], and we confirmed DAF-16 binding to *rheb-1* by chromatin IP (Fig 5C). Our genetic data also do not find any evidence for a contribution of PQM-1 to DAF-16 dependent germline tumor formation, as *pqm-1* RNAi in our hands did not modulate the tumor phenotype (S1 and S2 Tables). Moreover, *pqm-1* was reported to be expressed exclusively in the intestine, whereas our data suggest that hypodermal expression of all four downstream genes promotes cell proliferation in the germline. Together, these data suggest that *rsks-1*, *rheb-1*, *daf-15*, *and hpo-11* are candidates for direct transcriptional targets of R-Smad and/or DAF-16 to promote cell proliferation in the germline. Our results also indicate that DAF-16 can activate mTORC1 at the transcriptional level. This differs from the previous report that DAF-16 represses *daf-15* transcription in *daf-2(-)* mutant animals [26], suggesting a context dependent regulation of DAF-16 on mTORC1. We also showed that presence or absence of DAF-2 determines whether DAF-16 activates or represses transcription of mTORC1 components (Fig 5D). Similarly, as we will discuss below, presence or absence of DAF-2 also determines whether DAF-16 acts oncogenic or as a tumor suppressor. Such antagonistic effect of FOXO is also known from human tissues [47]. Finally, our results indicate that similar to the widely accepted tumorigenic activity of mTORC1 in other organisms, its *C*. *elegans* counterpart may fulfill a comparable role [48, 49].

### Antagonistic functions of DAF-16 in distinct tissues control germline proliferation under different physiological conditions

**Fig 7:**
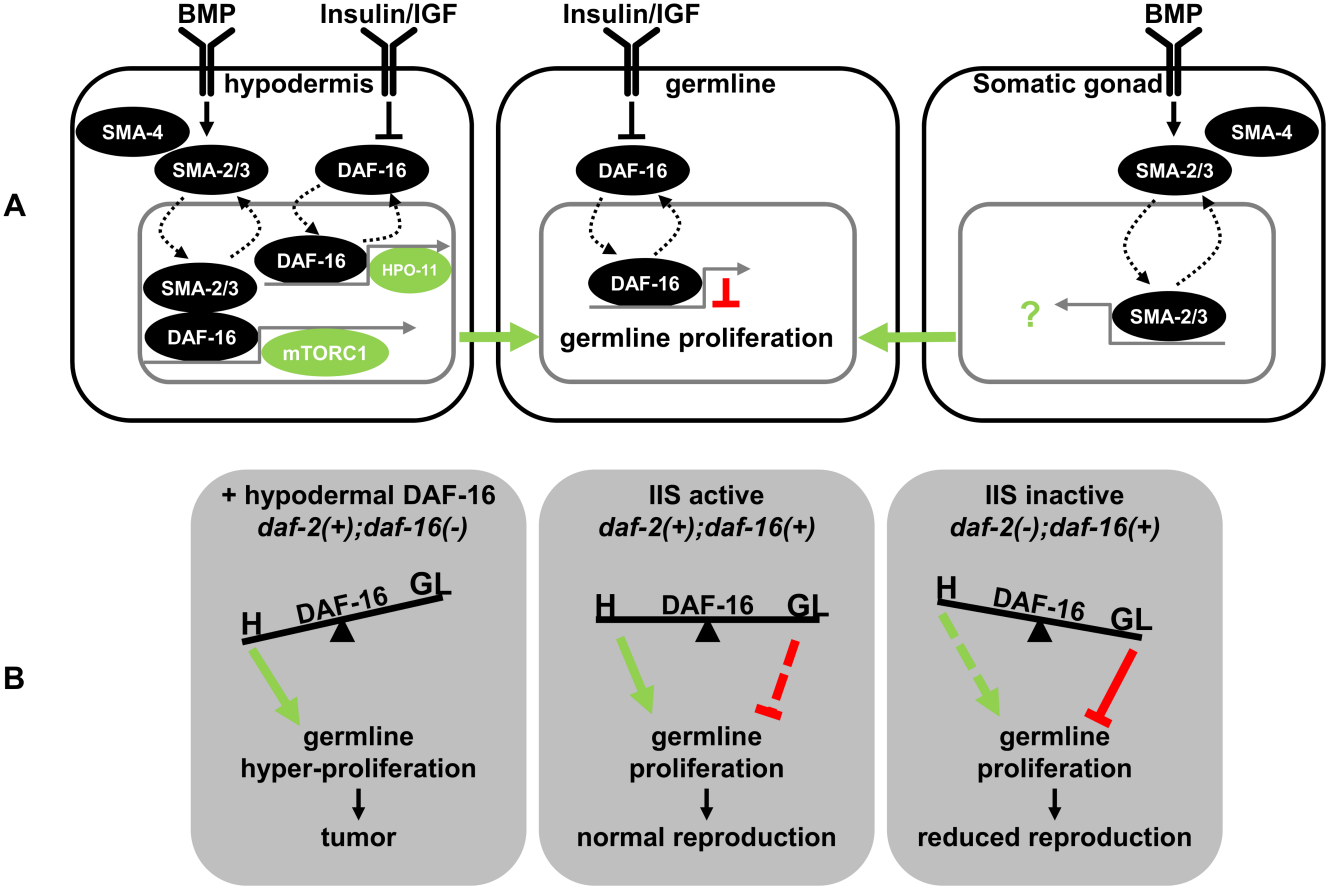
IIS balances anti-proliferating (tumor-suppressive) and pro-proliferating (oncogenic) activities across tissue boundaries. (A) Model of the cell-autonomous and non-cell-autonomous functions of DAF-16. IIS inhibits nuclear entry and activity of DAF-16/FOXO, BMP promotes nuclear entry and activates R-SMAD proteins SMA-2 and SMA-3. Both DAF-16 and SMA-2/3 have individual roles and regulate transcription of distinct target genes [19, 42, 50]. For example, BMP activity in the hypodermis controls body size, oocyte and germline quality maintenance independently of IIS. We currently have no evidence for germline activity of BMP signaling. In the hypodermis DAF-16 interacts with SMA-2/3 in the nucleus to activate mTORC1 at transcriptional level to promote germline proliferation (green arrow). DAF-16 also activates *hpo-11* transcription independent of BMP signaling to promote cell proliferation. In addition, BMP signaling in the somatic gonad also positively regulate germline proliferation, while DAF-16 in the germline has been proposed to inhibit cell proliferation (inhibitory activity in red) [5]. (B) Hypodermal and germline DAF-16 activities need to be tightly balanced to prevent hyperproliferation. Signaling activities of at least two tissues contribute to IIS homeostasis and may explain, why DAF-16 hyperactivity (in the hypodermis) may cause germline tumors in *daf-2(+)* background, whereas *daf-2(-)* (or starvation) decreases DAF-16 oncogenic potential, although in a simplistic view, it is thought to activate DAF-16.

The complexity of IIS signaling and DAF-16 activities in distinct tissues was highlighted by the observation of germline tumors in distinct genetic contexts. Mutant genetic backgrounds resulting in germline tumors were *shc-1;Is[daf-16*::*GFP]*, *shc-1;akt-1*, or *shc-1;daf-18;akt-1* [6]. Common denominator is that DAF-16 is active in all strains, and *shc-1* inactivation is a requirement for their solid phenotypic penetrance. However, both *shc-1;Is[daf-16*::*GFP]*, and *shc-1;akt-1* only resulted in tumors in the presence of *daf-2(+)*, and *daf-18*/PTEN(-) exacerbated *shc-1;akt-1* tumor penetrance, but did not cause germline proliferation defects in either *shc-1* or *akt-1* alone. These observations are, as we reported earlier [6], inconsistent with a simple model based on the canonical insulin signaling pathway, since *daf-2(+)* and *daf-18(-)* both are commonly known as negative regulators of DAF-16. Therefore, we hypothesize that down-regulation of *daf-2* to suppress the DAF-16 induced germline phenotype may predominantly affect the germline rather than somatic functions of IIS [6]. It has been shown that *daf-2* inactivation reduced mitotic rates of the germline stem cells via a cell-autonomous DAF-16 activity to arrest cell cycle [5]. We corroborated this result, since we counted a 40% reduction of the germline nuclei in the proliferative zone in *daf-2(e1370)*. This reduction was almost completely dependent on DAF-16 (S9 Fig), suggesting a conserved function of IIS to tie nutrition to cell cycle control, and a cell-autonomous function of DAF-16 in the germline to prevent tumorigenesis. Our results, in contrast, suggest a distinct, germline proliferation-promoting and non-cell-autonomous role of DAF-16 which acts in the hypodermis and becomes dominant when the balance between somatic and germline IIS is perturbed. Since hypodermal expression of a *daf-16* transgene in *daf-16* mutant animals was sufficient to increase the number of germ cell nuclei in the proliferative zone, these two antagonistic activities of DAF-16 in the hypodermis versus the germline should function in parallel (Fig 7). In well-fed animals, DAF-2 is considered to be active, and DAF-16 therefore should be inactive. Consistently, DAF-16::GFP fusion proteins in somatic tissues have been shown to localize mostly to the cytosol. However, a pool of active DAF-16 must still be present, since *daf-16* loss-of-function mutants can be phenotypically distinguished from *daf-16(+)* wild type animals due to their reduced number of proliferative germ cells (Fig 6A). Therefore, we propose that, in the presence of food, residual DAF-16 activities in both tissues are balanced with pro-proliferating hypodermal dominating over anti-proliferating germline DAF-16 to allow robust germline proliferation. These two opposing functions of DAF-16 allow a balanced response to diverse environmental challenges to ensure survival and optimal reproduction. Upon food limitation and subsequent reduction of IIS, DAF-16 activity in the germline becomes dominant and inhibits germline mitosis. Such different responses of somatic and germline cells, however, require differences in the composition of the IIS pathway in both tissues, for example, tissue-specific utilization of pathway components. Candidates mediating such differences are the AGC family kinases AKT-1, AKT-2, and SGK-1. Indeed, our previous experiments had already shown that the individual distribution of AKT kinases and SGK-1 differ between individual tissues, which might explain why *shc-1;akt-1* germline tumor are potently suppressed by RNAi down-regulation of either *akt-2* or *sgk-1* [6]. However, in certain genetic backgrounds, in which hypodermal DAF-16 activation dominates and this balance is lost, the consequence may be germline tumor.

### Interaction between FOXO and BMP signaling

FOXO transcription factors play an essential role in maintaining hematopoietic stem cells [51]. An important function of FOXO3a was also proposed in the maintenance of cancer stem cells that are responsible for the reoccurrence of chronic myeloid leukemia [52]. In addition, FOXO 3/4 has been shown to promote acute myeloid leukemia (AML) via inhibiting myeloid maturation and apoptosis [47]. Due to a critical role of the PI3K/Akt/mTOR signaling pathway in survival and growth of malignant cells, mTOR inhibitors like rapamycin are used in treating AML. We showed here that mTORC1 can be activated by DAF-16 and BMP signaling to promote germline tumor and it remains to be tested whether this phenotype is sensitive to rapamycin treatment. Even more interesting is the identification of *hpo-11* as a novel DAF-16 target gene, because its mammalian ortholog MADM (Mlf-1 adaptor molecule) physically interacts with myeloid leukemia factor 1 (Mlf1), which is involved in AML [53]. Interestingly, a recent publication had suggested that *Drosophila* MADM regulates the competition between germline stem cells and somatic cyst stem cells, for niche occupancy and also to control tumorigenesis [54]. This may suggest that the molecular mechanisms contributing to stem cell tumors that we identified here could be evolutionarily conserved. To our knowledge there is only one study suggesting that FOXO1/3 regulates follicle growth or death by interacting with the BMP pathways in granulosa cells [55]. It will be of great interest to test whether functional interaction between DAF-16 and BMP signaling is also conserved in *Drosophila* and in mammalian tumorigenesis, especially in AML.

## Materials and methods

Information about strains and constructs used is described in the S1 Materials and methods.

### Scoring disruption of the gonadal basement membrane

Scoring of disruption of the gonadal basement membrane at L3 larval stage was performed with MitoTracker staining which has been described to label basement membrane [56]. Basement membranes were stained by placing animals in a solution of 20 μM MitoTracker Red CMXRos (Molecular Probes) in M9 buffer at 23°C for 1 hr. The animals were allowed to recover for 30 min on NGM agar plate. The mitochondria staining with MitoTracker was photobleached using a 30 s exposure to fluorescence, leaving the basement membrane staining which is resistant to photobleaching.

For scoring disruption of the gonadal basement membrane in day one adults, animals were raised at 20°C and analyzed via DIC microscopy 24 hr after L4 stage. Germ cell outside of the gonad was indicator of gonad disruption. Per test at least thirty animals were examined and each test was performed at least three times.

### Quantification of the germ cell nuclei in L3 animals

For counting total numbers of the germ cell nuclei, the numbers of anti-PGL-1 positive cells were quantified at the mid-late L3 stage. Antibody staining was performed as described before [6]. For the whole worm staining, 1:200 anti-PGL-1 antibody was used (a gift from Dr. Susan Strome).

### Lifespan assay

Lifespan assays were initiated at the L4 larval stage. Synchronized animals were raised at 15°C prior to lifespan analysis. In order to inactivate *fer-15*, L4 animals were transferred to 25°C for the assays and examined every day. Animals that showed no response to touch were scored as dead. All of the lifespan assays were performed three times.

### Quantification of germ cell nuclei in the proliferative zone via DAPI staining

The ‘number of nuclei in the proliferative zone’ included all the germ nuclei between the distal tip and the transition zone of day one adult animals (24 hr after mid-L4 larval stage). For visualizing and counting germ cell nuclei in the proliferating zone of the gonad, dissected gonads were fixed in methanol and resuspended in PBST containing 0.1% Tween 20 and 2 μg/ml 4’,6-diamidino-2-phenylindole (DAPI) before microscopy. Z-stack images of animals were collected and the numbers of the germ cells were counted by using a ImageJ Cell Counter plug-in originally written by Kurt De Vos at the University of Sheffield, Academic Neurology.

### Yeast two-hybrid assay

The cDNAs of interaction partners were cloned into pGBKT7 and pGADT7 yeast vectors. Competent cells of the yeast strain AH109 were cultured at 30°C until OD_600_nm value 0.8–1.2. After centrifugation, the pellets were resuspended in 1 ml LiAc-TE solution (100 mM LiAc, 10 mM Tris, pH 7.4, 1 mM EDTA). The transformation was conducted by adding 10 μg boiled salmon sperm DNA (2 mg/ml), 500 μl PEG (50% PEG MG3350) and 1 μg of each plasmid DNA to 100 μl yeast aliquot. After incubation at 30°C for 15 min, 50 μl DMSO were added. After heat shocking at 42°C for 15 min, the cells were spread onto plates without Trp or Leu. The interaction was double determined by spreading the transformed yeast onto the two selective plates (-Leu/-Trp/-His with 3 mM 3-AT and -Leu/-Trp/-Ade).

### Microarray analyses

Total RNA was prepared from L3 animals raised at 20°C by using RNeasy Mini Kit (Qiagen, Venlo, The Netherlands). RNA integrity was analyzed by capillary electrophoresis using a Fragment Analyser (Advanced Analytical Technologies, Inc. Ames, IA). RNA samples had RNA quality numbers (RQN) between X and Y and were further processed with the Affymetrix WT Plus kit and hybridized to GeneChip *C*. *elegans* 1.0 ST arrays as described by the manufacturers. Partek Genomics Suite software was used for analysis (Partek Inc., St.Louis, MI). CEL files were imported including control and interrogating probes, pre-background adjustment was set to adjust for GC content and probe sequence, and RMA background correction was performed. Arrays were normalized using Quantile normalization and probe set summarization was done using Median Polish. Probe values were log2 transformed. For the statistical analysis of differentially expressed genes log-scale gene expression values were imported into R and analyzed using the limma package [57]. For GO term analysis we used g:Profiler (http://biit.cs.ut.ee/gprofiler)

### Quantification of mRNA level via Quantitative RT-PCR (q-PCR)

Total RNA was prepared from L3 animals raised at 20°C by using RNeasy Mini Kit (Qiagen, Venlo, The Netherlands). *act-4* was used as internal control. q-PCR primer sequences *daf-15* for: GACAACCGCAAGGAATTATGA, *daf-15* rev: CGCCGAGAAGTTGAAGGA, *hpo-11* for: CACACGGTTTTATGTGTCAGC, *hpo-11* rev: TTCCTGAACACCTTCTGCAAT, *rsks-1* for: TCCACCAAATGTTCGTGTTG, *rsks-1* rev: GTCGTTTTTCGCACTTGGA, *rheb-1* for GAAAATCGGCGTTGGTACTT, *rheb-1* rev: GGAACAACTTCTCGGGAAAAC. *act-4* for: CCACCATGTACCCAGGAATC, *act-4* rev: GTGGGGCGATGATCTTGA.

### Chromatin IP followed by q-PCR

ChIP was performed as described in Kaletsky et al. [58] by using anti-GFP antibody in wild type (untagged), *shc-1(ok198);Is[daf-16*::*GFP]*, *daf-2(e1370);Is[daf-16*::*GFP]* and a somatic exclusive *GFP*::*sma-3* strain (CS119 *sma-3(wk30)III;him-5(e1490)V;qcEx24[GFP*::*sma-3;rol-6]*). The relative fold change of qPCR was normalized to the intern negative control which was *daf-15* 3’UTR sequence.

q-PCR primer sequences: *P*_*sod*_*-3* for: ACAACAATGTGCTGGCCTTG, P_sod-3_ rev: AATGCATTTCGGGACGTTAG, *P*_*rheb-1*_ for: AATAACGCTTTCAACGCGGAG, P_rheb-1_ rev: ACCGTACCCAAGCAAACCTG, *P*_*hpo-11*_ for: CCCTTTGGCCGATTCTTGTC, P_hpo-11_ rev: GAGCCACATGAGACACACAC, *daf-15* 3’UTR for: AAAAGGCGCTTCATCATCCC, *daf-15* 3’UTR rev:CACATGAAATTGGTCCCCGC.

### Statistical analysis

GraphPad Prism 6.0 software (GraphPad Software Inc., San Diego, USA) was used to analyze the data. One way analysis of variance followed by Dunnett’s multiple comparison test was used to evaluate statistical significance of multiple groups of samples unless stated otherwise. For all statistical tests the 0.05 level of confidence was accepted as a significant difference.

## Supporting information

S1 Materials and Methods Information about strains and constructs used. (PDF)

S1 Fig Canonical *vs*. novel DAF-16 functions. Left side (grey): The canonical model of DAF-16 regulation proposes a linear pathway, in which active insulin receptor DAF-2 activates the three down-stream AGC kinases AKT-1, AKT-2 and SGK-1 [59]. AKT-1 and AKT-2 act redundantly and phosphorylate DAF-16 to prevent its nuclear localization and activation. The role of SGK-1 is debated, as different studies propose distinct SGK-1 functions either as DAF-16 inhibitor or activator [60]. DAF-18 and SHC-1 antagonize insulin signalling, thus eventually contributing to DAF-16 activation [20]. Active DAF-16 under reduced insulin signalling extends lifespan, enhances stress resistance and inhibits germline proliferation [5]. Right side (white): Qi et al. (2012) proposed a model according to which hypodermal DAF-16 activation opposes cell-autonomous DAF-16 that has been shown to act in the germline to inhibit germ cell proliferation [6]. Imbalance in DAF-16 inputs into the germline may cause germline tumor formation and reduce lifespan. *shc-1;akt-1* double inactivation induce germline tumor formation. In addition, *akt-2* or *sgk-1* downregulation prevent, whereas *daf-18(-)* increases the penetrance of *shc-1;akt-1* tumorous phenotype. It is currently not known whether AKT-2 and SGK-1 antagonize AKT-1 in the same or different tissues. *daf-2(-)* prevents tumor formation, likely because tumor-promoting DAF-16 activity is blocked, whereas cell-autonomous tumor-supressive function of DAF-16 is activated.
(TIF)

S2 Fig Representative pictures of *shc-1(ok198);Is[daf-16*::*GFP]* animals fed with *E*. *coli* HT115 carrying empty vector (L4440) or suppressor RNAi. (A) About 50% of the *shc-1;Is[daf-16*::*GFP]* animals are sterile due to tumorous germline, the remaining have strongly reduced brood sizes (typically <50 progeny). Therefore, embryos and larval progeny are rarely observed on plates. (B) Suppression of the germline tumor restored reproduction, as obvious from the accumulation of embryos and larval progeny on the plate. Black arrows: P0 adult animals, red arrows: F1 progeny.
(TIF)

S3 Fig Classification of gonadal basement membrane phenotype. Shown is a representative picture of wild type (intact) and mutant (intermediate or severe defect) gonadal basement membrane stained with MitoTracker. Arrows point to disruptions of gonadal basement membrane in L3 animals of the “intermediate” group. In severe cases, the basement membrane is so substantially disturbed, that the shape and organization of the gonad is no longer recognizable. Note that MitoTracker also stains basement membranes of the other tissues.
(TIF)

S4 Fig *sma-3* is expressed in the somatic gonad. The arrows in this representative picture points to an L3 somatic gonad cell expressing the *GFP*::*sma-3* transgene (*byEx1236[Psma-3*::*sma-3*::*GFP]*).
(TIF)

S5 Fig No interaction between SMA-2 and SMA-3 in yeast-two-hybrid assay. Yeast-two-hybrid interactions testing different combinations of bait and prey plasmids on two distinct selective media (labelled on top). SMA-2AD and SMA-3AD interact with DAF-16BD, FTT-2BD interacts with DAF-16AD (positive control). No interaction was observed between SMA-2 and SMA-3.
(TIF)

S6 Fig Microarray result. Heatmap (red/blue = high/low expression levels) of microarray results for the *shc-1;Is[daf-16*::*GFP] (bottom row)*, the *shc-1 daf-16 (middle)* and the *shc-1;sma-6;Is[daf-16*::*GFP] (top)* strains. Common target genes of DAF-16 and BMP signaling are found in clusters for which the coloring of the bottom row differs from that in the top row and in the middle row.
(TIF)

S7 Fig DAF-16 binds to promoters of *hpo-11*, *rheb-1*, *daf-15* and *rsks-1*. Wormbase (www.wormbase.org) gene models for *hpo-11*, *rheb-1*, *daf-15* and *rsks-1* are shown with modENCODE data from DAF-16 ChIP-seq experiments. The positions of primer sets for ChIP-qPCR and predicted DAF-16 binding sites [42, 61] are pointed in the promoter regions. Red line: DAF-16 binding element (DBE) GTAAAC/TAA or TTA/GTTTAC; black line: DAF-16 associate element (DAE); blue line: the predicted DAF-16 binding sites TGGAA/CAAT or ATTG/TTCCA.
(TIF)

S8 Fig DAPI staining of representative germlines of day one adult animals of the indicated genotypes. The white lines depict the borders of the transition zones between mitotic and meiotic germ cell nuclei. The white arrows indicate the position of the distal tip cells, the stem cell niche. Scale bar: 10 μm.
(TIF)

S9 Fig DAF-16 inhibits germline proliferation upon reduced IIS. The average numbers of germ cell nuclei in the proliferative zone are: wild-type: 249 ± 18 (n = 26); *daf-2(e1370)*: 155 ± 18 (n = 23); *daf-16(mu86);daf-2(e1370)*: 234 ± 24 (n = 19).
(TIF)

S1 Table Sterility. (PDF)

S2 Table Defects in gonadal integrity. (PDF)

S3 Table Summary of lifespan. (PDF)

S4 Table Summary of dauer formation at 25°C. (PDF)

S5 Table Summary of body length. (PDF)

S6 Table Transgenic animals generated for this work. (PDF)

S7 Table Enriched GO terms for genes which are upregulated both by DAF-16 and BMP signaling in *shc-1;Is[daf-16*::*GFP]* L3 animals. (PDF)

S8 Table Summary of quantification of relative mRNA level. (PDF)

S9 Table *daf-15* and *rsks-1* primers tested for ChIP-qPCR. (PDF)

S10 Table Numbers of germ nuclei in the proliferation zone of day one adults. (PDF)
