# Supplementary material for: C. elegans DAF-16/FOXO interacts with TGF-ß/BMP signaling to induce germline tumor formation via mTORC1 activation
Source: PLoS Genet. 2017 May 26;13(5):e1006801. doi: 10.1371/journal.pgen.1006801 (PMC5467913; doi:10.1371/journal.pgen.1006801)
Supplement: S1 Materials and Methods — (PDF) [file pgen.1006801.s001.pdf]

Supplemental Materials and Methods

Strains

The strains used are shown as follow: wild-type N2 Bristol, BR5082 *shc-1(ok198);zIs356[daf-16::GFP]*, CF1038 *daf-16(mu86)*, CB1370 *daf-2(e1370)*, BR5054 *daf-16(mu86);daf-2(e1370)*, LT186 *sma-6(wk7),* BR6509 *shc-1(ok198);sma-6(wk7);zIs356[daf-16::GFP],* BR6732 *shc-1(ok198);dbl-1(nk3);zIs356[daf-16::GFP],* BR6699 *shc-1(ok198);sma-2(e502);zIs356[daf-16::GFP],* BR6734 *shc-1(ok198);sma-3(e491);zIs356[daf-16::GFP],* BR6515 *shc-1(ok198);sma-9(ok1628);zIs356[daf-16::GFP]*, DH26 *fer-15(b26),* BR4631 *fer-15(b26);daf-2(e1370),* BR6514 *sma-6(wk7);daf-2(e1370),* BR6560 *daf-16(mu86);sma-6(wk7);daf-2(e1370),* BR5358 *shc-1(ok198);byEx800[Pdaf-16::daf-16(4A)::GFP;rol-6]*, BR6715 *shc-1(ok198);sma-6(wk7);byEx800[Pdaf-16::daf-16(4A)::GFP;rol-6]*, BR5875 *shc-1(ok198) rrf-1(ok589);zIs356[daf-16::GFP],* CS119 *sma-3(wk30)III;him-5(e1490)V;qcEx24[GFP::sma-3;rol-6], Ex[Peif-4::GFP]* as negative GFP control for DAF-16/SMA-3 IP, CF1553 *muIs84[Psod-3::GFP],* BR6621 *daf-2(e1370);muIs84[Psod-3::GFP],* BR6659 *daf-16(mu86);daf-2(e1370);muIs84[Psod-3::GFP],* BR6619 *sma-6(wk7);daf-2(e1370);muIs84[Psod-3::GFP],* BR6960 *daf-16(mu86);byIs217[Pdpy-7::daf-16(4A)::GFP]*, BR7208 *daf-16(mu86);byEX1351[Pdpy-7::daf-16::GFP],* BR7354 *daf-16(mu86);byEx1391[Pges-1::daf-16::GFP]*, BR7348 *daf-16(mu86);byEx1385[Punc-119::daf-16::GFP]*, BR7351 *daf-16(mu86);byEx1388[Pmyo-3::daf-16::GFP]*, BR7729 *daf-16(mu86);byEx1322[Pdpy-7::sma-6]*, BR7137 *sma-6(wk7);byEx1322[Pdpy-7::sma-6]*, BR7767 *sma-6(wk7);byEx1524[Pmyo-2::sma-6],* BR7220 *sma-*

6(wk7);byEx1347[Pges-1::sma-6], BR7731 *sma-6;byEx1351[Pdpy-7::DAF-16::GFP],* NR222 *rde-1(ne219);kzIs9[Plin-26::rde-1],* BR7820 *daf-16(mu86);rde-1(ne219);kzIs9[Plin-26::rde-1],* BR7821 *sma-6(wk7);rde-1(ne219);kzIs9[Plin-26::rde-1]*.

To generate transgenic animals carrying *sma-6* or *daf-16,* the corresponding constructs were injected into *shc-1(ok198);sma-6(wk7);zIs356[daf-16::GFP]*, *sma-6(wk7) or daf-16(mu86)* (for allele numbers, see Supplemental Table S5). 20 ng/µl *Pmyo-2::mCherry or Pmyo-2::CFP* was used as co-injection marker. GFP expression of the transgenic animals was confirmed by using fluorescent microscopy.

Plasmids

All constructs, if not mentioned otherwise, were generated with the pEGFP-N1 vector (Clontech). The *Psma-6::sma-6* (pBY3801) plasmid was generated by inserting a 5.2 kb genomic fragment including 2.3 kb promoter region of *sma-6* at the *PstI/AgeI* sites. Primers used for cloning: forward primer: CCCCTGCAGATTGTCATTTGAAATGTGGACGGAC; reverse primer: CCCACCGGTTTAAGATTGATTGGTGGCTGACTC. To express *sma-6* in the hypodermis (pBY3721 *Pdpy-7::sma-6*); pharynx (pBY3723 *Pmyo-2::sma-6*) and intestine (pBY3722 *Pges-1::sma-6*), respectively, *dpy-7*, *myo-2*, and *elt-2* promoters were inserted at the *Eco*47III/*Hind*III, *Nhe*I/*Apa*I and *Bgl*II/*Eco*RI sites to drive the expression of *sma-6* cDNA, which was inserted at the *Sma*I/*Age*I sites.

Yeast two-hybrid protein interaction study was performed using the MATCHMAKER GAL4 Two-Hybrid System 3 according to the manufacturer's instructions (Clontech). DAF-16 and SMA-3 were fused to GAL4 activation domain (pGADT7) and GAL4 DNA

binding domain (pGBKT7) at the *SfiI/Bam*HI sites while SMA-2 and FTT-2 were inserted into pGADT7 and pGBKT7 vector at the *Nde*I/SmaI sites.

To express recombinant DAF-16, SMA-2 and SMA-3 in HEK293 cells, *daf-16a* cDNA was cloned into pcDNA6A::V5-6His (pBY3924) vector at the *NheI/NotI* sites, *sma-2* cDNA was into pEGFP-N1vector at *SalI/NheI* sites (pBY3923) and *sma-3* cDNA into pEGFP-C1 vector at *KpnI/SmaI* sites (pBY3932).
