# Supplementary material for: C. elegans DAF-16/FOXO interacts with TGF-ß/BMP signaling to induce germline tumor formation via mTORC1 activation
Source: PLoS Genet. 2017 May 26;13(5):e1006801. doi: 10.1371/journal.pgen.1006801 (PMC5467913; doi:10.1371/journal.pgen.1006801)
Supplement: S1 Table — (PDF) [file pgen.1006801.s011.pdf]

S1 Table. Sterility

strains	sterile	n
N2	0%	90
shc-1(ok198);Is[daf-16::GFP]	49%	90
+*sma-6(wk7)*	0%	121
+*dbl-1(nk3)*	0%	101
+*sma-2(e502)*	0%	142
+*sma-3(e491)*	0%	131
+*sma-9(ok1628)*	0%	175
+L4440	51%	100
+*pqm-1*(RNAi)	53%	98
***shc-1(ok198);Is[daf-16::GFP]* (L4440)**	47%	90
+ *rsks-1* RNAi	1%	106
+ *hpo-11* RNAi	0%	123
+ *sma-6* RNAi	0%	95
***shc-1(ok198) rrf-1(ok589);Is[daf-16::GFP]* (L4440)**	14%	94
+ *sma-6* RNAi	15%	98

n: number of examined animals.
This table is related to the main Fig 1.
