# Supplementary material for: C. elegans DAF-16/FOXO interacts with TGF-ß/BMP signaling to induce germline tumor formation via mTORC1 activation
Source: PLoS Genet. 2017 May 26;13(5):e1006801. doi: 10.1371/journal.pgen.1006801 (PMC5467913; doi:10.1371/journal.pgen.1006801)
Supplement: S2 Table — (PDF) [file pgen.1006801.s012.pdf]

S2 Table. Defects in gonadal integrity

Genotype	% day one adult with disrupted gonad	SD	n	P Value
N2 wild-type	0.0		90	
shc-1(ok198);Is[daf-16::GFP]	98.9	1.9	90	
+*sma-6(wk7)*	24.3	11.2	121	<0.0001[a]
+*dbl-1(nk3)*	43.9	18.7	101	<0.0001[a]
+*sma-2(e502)*	3.2	3.9	142	<0.0001[a]
+*sma-3(e491)*	17.2	18.3	131	<0.0001[a]
+*sma-9(ok1628)*	39.5	3.7	96	<0.0001[a]
shc-1(ok198);byEx800[daf-16(4A)::GFP]	57.0	10.5	90	
+*sma-6(wk7)*	5.1	4.3	108	<0.0001[b]
***shc-1(ok198) rrf-1(ok589);Is[daf-16::GFP]* (L4440)**	84.1	2.9	95	
+*sma-6* RNAi	78.5	7.5	130	0.2664[c]
***shc-1(ok198);Is[daf-16::GFP]* (L4440)**	100.0		96	
+*sma-6* RNAi	40.2	6.7	95	<0.0001[d]
+*daf-1* RNAi	98.9	1.9	90	
+*daf-4* RNAi	97.8	1.5	90	
+*rsks-1* RNAi	76.9	5.1	106	<0.0001[d]
+*hpo-11* RNAi	62.0	4.3	123	<0.0001[d]
+*pqm-1* RNAi	100		90	

n: Number of examined animals. P values were relative to: a: *shc-1(ok198);Is[daf-16::GFP]*; b: *shc-1(ok198);byEx800[daf-16(4A)::GFP]*; c: *shc-1(ok198)-1 rrf-1(ok589);Is[daf-16::GFP]* (L4440); d: *shc-1(ok198);Is[daf-16::GFP]*(L4440); e: *shc-1(ok198);sma-6(wk7);Is[daf-16::GFP].*
This table is related to the main Fig 1 and 2.
