# Supplementary material for: C. elegans DAF-16/FOXO interacts with TGF-ß/BMP signaling to induce germline tumor formation via mTORC1 activation
Source: PLoS Genet. 2017 May 26;13(5):e1006801. doi: 10.1371/journal.pgen.1006801 (PMC5467913; doi:10.1371/journal.pgen.1006801)
Supplement: S3 Table — (PDF) [file pgen.1006801.s013.pdf]

S3 Table. Summary of lifespan

Genotype	Mean lifespan at 25°C (days)	Number (n) of examined animals
fer-15(b26) + L4440	13.4	87
fer-15(b26)+ *sma-6* RNAi	12.8	130
fer-15(b26);daf-2(e1370) + L4440	25.2	90
fer-15(b26);daf-2(e1370) + *sma-6* RNAi	27.2	84

This table is related to the main Fig 2.
