# Supplementary material for: C. elegans DAF-16/FOXO interacts with TGF-ß/BMP signaling to induce germline tumor formation via mTORC1 activation
Source: PLoS Genet. 2017 May 26;13(5):e1006801. doi: 10.1371/journal.pgen.1006801 (PMC5467913; doi:10.1371/journal.pgen.1006801)
Supplement: S4 Table — (PDF) [file pgen.1006801.s014.pdf]

S4 Table. Summary of dauer formation at 25°C

Genotype	% dauer at 25°C	Number (n) of examined animals
daf-2(e1370)	97%	217
sma-6(wk7)	0%	236
sma-6(wk7);daf-2(e1370)	98%	252

This table is related to the main Fig 2.
