# Supplementary material for: C. elegans DAF-16/FOXO interacts with TGF-ß/BMP signaling to induce germline tumor formation via mTORC1 activation
Source: PLoS Genet. 2017 May 26;13(5):e1006801. doi: 10.1371/journal.pgen.1006801 (PMC5467913; doi:10.1371/journal.pgen.1006801)
Supplement: S5 Table — (PDF) [file pgen.1006801.s015.pdf]

S5 Table. Summary of body length

Strains	Body length±SD (mm)	Number (n) of examined animals
N2	1.10±0.09	16
daf-2(e1370)	1.22±0.06	13
daf-16(mu86);daf-2(e1370)	1.08±0.06	19
sma-6(wk7)	0.70±0.06	14
sma-6(wk7);daf-2(e1370)	0.94±0.06	10
daf-16(mu86);sma-6(wk7);daf-2(e1370)	0.59±0.04	21

n: numbers of examined animals.
This table is related to the main Fig 2.
