# Supplementary material for: C. elegans DAF-16/FOXO interacts with TGF-ß/BMP signaling to induce germline tumor formation via mTORC1 activation
Source: PLoS Genet. 2017 May 26;13(5):e1006801. doi: 10.1371/journal.pgen.1006801 (PMC5467913; doi:10.1371/journal.pgen.1006801)
Supplement: S6 Table — (PDF) [file pgen.1006801.s016.pdf]

S6 Table. Transgenic animals generated for this work

genotype / transgene	expression in:
***shc-1;sma-6;zIs356[daf-16::GFP*] +**	
byEx1248[Pelt-2::sma-6; Pmyo-2::mCherry]	intestine
byEx1251[Pmyo-2::sma-6; Pmyo-2::mCherry]	pharynx
byEx1486[Pfos-1:.sma-6;Pmyo-2::mCherry]	somatic gonad
byEx1527[Pfos-1:.sma-6;Pdpy-7::sma-6;Pmyo-2::mCherry]	somatic gonad and hypodermis
byEx1530[Pfos-1:.sma-6;Pmyo-2::sma-6;Pelt-2:.sma-6;Pmyo-2::mCherry]	somatic gonad, intestine and pharynx
***sma-6(wk7)* +**	
byEx1460[Pfos-1::sma-6::GFP;Pmyo-2::mCherry]	somatic gonad
byEx1524[Pmyo-2::sma-6;;Pmyo-2::CFP]	pharynx
byEx1347[Pelt-2::sma-6;Pmyo-2::CFP]	intestine
byEx1322[Pdpy-7::sma-6; Pmyo-2::CFP]	hypodermis
***daf-16(mu86)* +**	
byEx1351[Pdpy-7::daf-16::GFP; Pmyo-2::CFP]	hypodermis
byEx1385[Punc-119::daf-16::GFP;Pmyo-2::CFP]	neuron
byEx1388[Pmyo-3::daf-16::GFP;Pmyo-2::CFP]	muscle
byEx1391[Pges-1::daf-16::GFP;Pmyo-2::CFP]	intestine
byIs217[Pdpy-7::daf-16(4A)::GFP; Pmyo-2::CFP]	hypodermal nucleus

This table is related to the main Fig 3 and 5.
