# Supplementary material for: C. elegans DAF-16/FOXO interacts with TGF-ß/BMP signaling to induce germline tumor formation via mTORC1 activation
Source: PLoS Genet. 2017 May 26;13(5):e1006801. doi: 10.1371/journal.pgen.1006801 (PMC5467913; doi:10.1371/journal.pgen.1006801)
Supplement: S7 Table — (PDF) [file pgen.1006801.s017.pdf]

S7 Table. Enriched GO terms for genes which are upregulated both by DAF-16 and BMP signaling in *shc-1;Is[daf-16::GFP]* L3 animals

**Gene Ontology Category**	Gene count	P-value
**Cell signaling**		
phosphoprotein phosphatase activity	28	8,87E-11
protein kinase activity	36	1,59E-05
**Cell cycle**		
cell cycle process	30	3,57E-06
nuclear division	21	2,38E-04
cell division	18	8,25E-03
condensed chromosome	33	9,18E-03
**Reproduction**		
single organism reproductive process	39	3,18E-08
P granule	11	7,47E-04
**Energy pathway,**		
ATP binding	52	1,15E-03
**Metabolism**		
Metabolic pathways	20	2,50E-03
Fatty acid metabolism	5	5,93E-03
**Other**		
cytoskeleton	50	1,86E-16
Apoptosis - multiple species	3	5,84E-03
mRNA 3'-UTR binding	6	6,07E-03
Nitrogen metabolism	3	7,72E-03

This table is related to the main Fig 4.
