# Supplementary material for: C. elegans DAF-16/FOXO interacts with TGF-ß/BMP signaling to induce germline tumor formation via mTORC1 activation
Source: PLoS Genet. 2017 May 26;13(5):e1006801. doi: 10.1371/journal.pgen.1006801 (PMC5467913; doi:10.1371/journal.pgen.1006801)
Supplement: S8 Table — (PDF) [file pgen.1006801.s018.pdf]

S8 Table. Summary of quantification of relative mRNA level

hpo-11	N2	*shc-1;Is[daf-16::GFP]*	*daf-16 shc-1*	*shc-1;sma-6;Is[daf-16::GFP]*
Test 1	1.0	3.2	1.02	2.71
Test 2	1.0	2.38	1.08	1.06
Test 3	1.0	2.58	1.41	2.13
Average	1.0	2.72	1.17	1.97
SEM	0	0.25	0.12	0.48
P-Value			0.0049	0.2374
rheb-1				
Test 1	1.0	2.59	0.40	1.33
Test 2	1.0	2.60	1.12	1.45
Test 3	1.0	2.64	1.07	1.97
Average	1.0	2.61	0.85	1.58
SEM	0	0.02	0.23	0.20
P-Value			0.0014	0.0065
daf-15				
Test 1	1.0	2.99	0.47	0.006
Test 2	1.0	4.41	0.52	0.43
Test 3	1.0	4.31	0.97	0.34
Average	1.0	3.90	0.65	0.26
SEM	0	0.46	0.16	0.13
P-Value			0.0026	0.0016
rsks-1				
Test 1	1.0	3.34	1.06	1.47
Test 2	1.0	2.38	0.68	0.17
Test 3	1.0	2.23	1.23	0.81
Average	1.0	2.65	0.99	0.82
SEM	0	0.35	0.16	0.38
P-Value			0.0124	0.0231

hpo-11	N2	*daf-2*	*daf-16;daf-2*
Test 1	1.0	1.83	1.06
Test 2	1.0	2.27	1.00
Test 3	1.0	3.01	1.06
Average	1.0	2.37	1.04
SEM	0	0.37	0.02
P-Value			0.018
rheb-1			
Test 1	1.0	1.82	1.88
Test 2	1.0	0.87	1.18
Test 3	1.0	1.55	1.25
Average	1.0	1.41	1.43
SEM	0	0.28	0.22
P-Value			0.951
daf-15			
Test 1	1.0	0.07	0.35
Test 2	1.0	0.28	0.40
Test 3	1.0	0.10	0.56
Average	1.0	0.15	0.44
SEM	0	0.06	0.06
P-Value			0.034
rsks-1			
Test 1	1.0	0.46	0.60
Test 2	1.0	0.74	0.68
Test 3	1.0	0.63	0.74
Average	1.0	0.61	0.67
SEM	0	0.08	0.04

P-Value	0.525

*The P values were calculated by comparing *shc-1;Is[daf-16::GFP]* with *daf-16 shc-1* or *shc-1;sma-6;Is[daf-16::GFP]*, or *daf-2* with *daf-16;daf-2*, respectively.

This table is related to the main Fig 5.
