# Supplementary material for: C. elegans DAF-16/FOXO interacts with TGF-ß/BMP signaling to induce germline tumor formation via mTORC1 activation
Source: PLoS Genet. 2017 May 26;13(5):e1006801. doi: 10.1371/journal.pgen.1006801 (PMC5467913; doi:10.1371/journal.pgen.1006801)
Supplement: S9 Table — (PDF) [file pgen.1006801.s019.pdf]

S9 Table. *daf-15* and *rsks-1* primers tested for ChIP-qPCR

daf-15 primers	forward primers	reverse primers
1	CACTTGCACGAATCACGTCT	TCGGCAAAAAGTGCAAAAACC
2	AACGTTGCGAAACCCGTAAA	TCCGTTGCACTTTTTGTCTCA
3	TGCATGAGTCATCCCACAAAGT	GCTGTGATTCAGTGCTGCG
4	AGCACTGAATCACAGCCAAT	CGGGTTTCGCAACGTTTTCAT
5	TGCATGAGTCATCCCACAAAG	TGTGATTCAGTGCTGCGTAT
6	GATGAAAACGTTGCGAAACCC	GCGCTCCGTTGCACTTTT
7	ATGGACCTGGATGTATGCGT	CAATGATGTTCTGTTGTCACTGT
8	CCACCTGGTTGTGTTTGCAC	GCCTTTGGATACATTCGACGC
9	ATTTAAATTCCAGAGCTCCACCTG	ATACATCCAGGTCCATGGCAG
10	GCTCCACCTGGTTGTGTTTG	AAGCCTTTGGATACATTCGACG
rsks-1 primers	forward primers	reverse primers
1	AACCATGCGAAAAAGCGTGA	TCTCTCGTTTTTCACTCCCCC
2	ACTCAAAACCGCCGAGAAAC	AGCTTTGCTTGCCGATTTCA
3	TTTCGCGTTTTTACGGGCTT	TCGTGTCAAGACCAGGGACA
4	CTCAAAACCGCCGAGAAACC	GTACACCCGGTCGTGTCAAG
5	TTTGGAATTTCGCAACGACGA	AAGCACACAAATCGTGGCAAA
6	CGGGAGAGGGGTCGAAAAAC	GTCGTTGCGAAATTCCAAAGTG
7	TTTGGAATTTCGCAACGACG	CACACAAATCGTGGCAAAAACG
8	CCAAATTCCTTGTTGCTTCCCC	GCTTTCACCTTGACAACGCC
9	CTCCCTTTGTAGTCTCGCCG	TCTCTGTCGCTTGTACTCTGAC
10	TTCACACGCGTAAACAAGCA	TTCGGCGAGACTACAAAGGG

This table is related to the main Fig 5.
