# Supplementary material for: C. elegans DAF-16/FOXO interacts with TGF-ß/BMP signaling to induce germline tumor formation via mTORC1 activation
Source: PLoS Genet. 2017 May 26;13(5):e1006801. doi: 10.1371/journal.pgen.1006801 (PMC5467913; doi:10.1371/journal.pgen.1006801)
Supplement: S10 Table — (PDF) [file pgen.1006801.s020.pdf]

S10 Table. Numbers of germ nuclei in the proliferation zone of day one adults

Genotype/transgenes/RNAi	Number of germ nuclei	SEM	n	P-Value
**N2**	248.9	3.6	26	< 0.0001
daf-16(mu86)	201.6	4.0	37	
+ hypodermal *daf-16*	249.7	6.5	27	< 0.0001
+ hypodermal nuclear *daf-16*	238.7	6.6	32	< 0.0001
+ intestinal *daf-16*	233.7	6.0	25	< 0.0001
+ muscular *daf-16*	200.0	8.1	24	0.9999
+ neuronal *daf-16*	208.8	5.2	21	0.9313
+ hypodermal *sma-6*	188.0	7.9	22	0.4326
sma-6(wk7)	90.6	2.9	26	
+ somatic gonadal *sma-6*	194.5	7.0	22	< 0.0001
+ hypodermal *sma-6*	118.6	5.0	27	< 0.0001
+ pharyngeal *sma-6*	84.0	2.6	29	0.8915
+ intestinal *sma-6*	94.7	3.5	18	0.9148
+ hypodermal *daf-16*	81.0	3.5	19	0.6504
***rde-1;Is[Plin-26::rde-1]*+L4440**	227.3	3.0	26	
+ *daf-15* RNAi	194.0	5.9	21	< 0.0001
+ *rheb-1* RNAi	201.1	3.0	27	< 0.0001
+ *rsks-1* RNAi	171.7	4.4	22	< 0.0001
+ *hpo-11* RNAi	201.0	3.2	26	< 0.0001
***daf-16;rde-1;Is[Plin-26::rde-1]*+L4440**	207.3	6.4	25	
+ *daf-15* RNAi	206.0	5.1	23	0.9998
+ *rheb-1* RNAi	211	5.5	21	0.9680
+ *rsks-1* RNAi	177.5	3.9	25	0.0005
+ *hpo-11* RNAi	192.0	5.8	25	0.1450

Genotype/transgenes/RNAi	Number of germ nuclei	SEM	n	P-Value

***sma-6;rde-1;Is[Plin-26::rde-1]*+L4440**	93.9	4.1	19	
+ *daf-15* RNAi	99.5	3.1	27	0.6554
+ *rheb-1* RNAi	100.9	3.6	26	0.4730
+ *rsks-1* RNAi	77.4	2.6	25	0.0082
+ *hpo-11* RNAi	98.6	4.3	23	0.7916

This table is related to the main Fig 6.
Genotypes of the transgenic animals are summarized in the S6 Table.
